# Anti-Allergic Inflammatory Activity of Interleukin-37 Is Mediated by Novel Signaling Cascades in Human Eosinophils

**DOI:** 10.3389/fimmu.2018.01445

**Published:** 2018-06-22

**Authors:** Jing Zhu, Jie Dong, Lu Ji, Peiyong Jiang, Ting Fan Leung, Dehua Liu, Lai Guan Ng, Miranda Sin-Man Tsang, Delong Jiao, Christopher Wai-Kei Lam, Chun-Kwok Wong

**Affiliations:** ^1^Department of Chemical Pathology, The Chinese University of Hong Kong, Prince of Wales Hospital, Hong Kong, Hong Kong; ^2^Department of Paediatrics, The Chinese University of Hong Kong, Prince of Wales Hospital, Hong Kong, Hong Kong; ^3^Institute of Chinese Medicine, State Key Laboratory of Phytochemistry and Plant Resources in West China, The Chinese University of Hong Kong, Hong Kong, Hong Kong; ^4^School of Chinese Medicine, The Chinese University of Hong Kong, Hong Kong, Hong Kong; ^5^Singapore Immunology Network, Singapore, Singapore; ^6^State Key Laboratory of Quality Research in Chinese Medicines, Macau Institute for Applied Research in Medicine and Health, Macau University of Science and Technology, Taipa, Macau

**Keywords:** allergic asthma, eosinophils, interleukin-37, signal transduction, transcriptional profile

## Abstract

IL-1 family regulatory cytokine IL-37b can suppress innate immunity and inflammatory activity in inflammatory diseases. In this study, IL-37b showed remarkable *in vitro* suppression of inflammatory tumor necrosis factor-α, IL-1β, IL-6, CCL2, and CXCL8 production in the coculture of human primary eosinophils and human bronchial epithelial BEAS-2B cells with the stimulation of bacterial toll-like receptor-2 ligand peptidoglycan, while antagonizing the activation of intracellular nuclear factor-κB, PI3K–Akt, extracellular signal-regulated kinase 1/2, and suppressing the gene transcription of allergic inflammation-related *PYCARD, S100A9*, and *CAMP* as demonstrated by flow cytometry, RNA-sequencing, and bioinformatics. Results therefore elucidated the novel anti-inflammation-related molecular mechanisms mediated by IL-37b. Using the house dust mite (HDM)-induced humanized asthmatic NOD/SCID mice for preclinical study, intravenous administration of IL-37b restored the normal plasma levels of eosinophil activators CCL11 and IL-5, suppressed the elevated concentrations of Th2 and asthma-related cytokines IL-4, IL-6, and IL-13 and inflammatory IL-17, CCL5, and CCL11 in lung homogenate of asthmatic mice. Histopathological results of lung tissue illustrated that IL-37b could mitigate the enhanced mucus, eosinophil infiltration, thickened airway wall, and goblet cells. Together with similar findings using the ovalbumin- and HDM-induced allergic asthmatic mice further validated the therapeutic potential of IL-37b in allergic asthma. The above results illustrate the novel IL-37-mediated regulation of intracellular inflammation mechanism linking bacterial infection and the activation of human eosinophils and confirm the *in vivo* anti-inflammatory activity of IL-37b on human allergic asthma.

## Introduction

According to the Global Burden of Diseases Study 2015, it was estimated that about 358 million people worldwide were suffering from asthma, with 0.40 million deaths in 2015 (1). The prevalence of asthma has continued to rise, with a higher annual incidence in children than in adults. Asthma can be classified into allergic asthma and non-allergic asthma. Most children and half of adult patients suffer from allergic asthma which is distinguished from non-allergic asthma, due to the presence of elevated serum immunoglobulin E (IgE, total and specific) and provocation by inhaled or ingested allergens such as pollen, dust mite, mold, animal dander, and peanut (2, 3).

Among one of the most prevalent chronic respiratory diseases of the conducting airways, allergic asthma is clinically characterized by reversible bronchial constriction and airway hyper-responsiveness (AHR), often associated with elevated plasma IgE concentration. Increased differentiated CD4^+^ T helper type (Th)2 lymphocytes originated from bronchial tissues and draining lymph nodes secrete allergy-related Th2 pro-inflammatory cytokines such as IL-4, IL-5, and IL-13, thereby contributing to the pathogenesis and exacerbation of allergic asthma (4–6). It has been shown that respiratory bacterial and viral infection can provoke allergic inflammation in allergic asthma (7, 8). The innate immune system actually recognizes infection through pattern-recognition receptors, including toll-like receptors (TLRs), NOD-like receptors, and RIG-I-like receptor, which detect conserved microbial components called pathogen-associated molecular patterns (PAMPs) including TLR2 ligand peptidoglycan (PGN) (9).

Eosinophils are crucial effector cells of allergic inflammation that accumulate and infiltrate in local inflammatory tissues upon the mediation of the specific eosinophil chemokine CCL11, and intercellular adhesion molecule-1 on epithelial cells (10). Allergen-reactive Th2 lymphocytes are important for the induction and maintenance of allergic inflammation (11). Cytokines and chemokines released by Th2 cells (IL-4, IL-5, IL-6, IL-9, IL-10, and IL-13) and those induced by other cell types in response to Th2 cytokines (CCL11, transforming growth factor/TGF-β, and IL-11) mediate most of the pathophysiological mechanisms in allergic reaction such as the recruitment, activation, and delayed apoptosis of eosinophils, and degranulation to release granular toxic proteins from eosinophils (11). In allergic airway inflammation, activated eosinophils release cytotoxic cationic proteins such as eosinophilic cationic protein (ECP) to cause tissue damage and airway hyper-reactivity (12). Our previous publications have elucidated the underlying intracellular signaling mechanisms by which the interaction of eosinophils and epithelial cells can induce the secretion of inflammatory cytokines, cysteinyl leukotrienes, and eosinophilic degranulation for ECP release in allergic asthma (13–16).

Regulatory cytokine IL-37, the seventh interleukin factor of IL-1 family (IL-1F7), can downregulate systemic and local inflammation by suppressing the production of pro-inflammatory mediators in both innate and adaptive immunity (17). IL-37 can be expressed in various human tissues such as skin, tonsils, esophagus, placenta, breast, prostate, and colon (18), and induced by TLR ligands and inflammatory cytokines IL-1β, tumor necrosis factor (TNF)-α, and interferon-γ in peripheral blood mononuclear cells (PBMCs) and dendritic cells (DCs) (19). IL-37 can suppress the production of various pro-inflammatory cytokines, including IL-1α, IL-1β, IL-6, IL-12, granulocyte colony-stimulating factor, granulocyte-macrophage colony-stimulating factor, and TNF-α, probably by forming an intracellular functional complex with transcription factorSmad-3 to regulate relevant gene transcription (17). Moreover, IL-37 can be expressed by regulatory T (Treg) cells to enhance the expression of anti-inflammatory TGF-β and IL-10, Foxp3, and cytotoxic T-lymphocyte associated antigen-4 to promote the immunosuppressive activity of human Treg cells (20). In addition, extracellular IL-37 can bind to IL-18-binding protein, IL-18Rα, and β-chain, thereby inhibiting the pro-inflammatory activity of IL-18 (17, 21). Apart from being a natural inhibitor of innate immunity, IL-37 can inhibit DC activation to regulate adaptive immunity (17, 22, 23). IL-37 binds to the IL-18 receptor but recruits the orphan IL-1R8 to act as an inhibitor to exhibit its multifaceted anti-inflammatory activity, *via* the regulation of cellular adhesion and migration, and intracellular mechanism in murine splenic lymphocytes (17, 24). IL-37 can also ameliorate experimental asthma by Th2 suppression that is independent of IL-18 signaling (25). Although no mouse homolog has yet been found, human IL-37 has been reported to be an *in vivo* suppressor of inflammation in mice (17). Using murine experiments, IL-37 has been shown to play immunoregulatory roles in myocardial infarction, rheumatoid arthritis (RA), diabetes, allergic asthma, and fungal infections (26–30). Clinical studies have demonstrated the downregulated production of IL-37 of human PBMC in allergic asthmatics and decreased IL-37 level in induced sputum with negative correlation with disease severity of asthma (29, 31). The *ex vivo* lipopolysaccharide (LPS)-induced release of pro-inflammatory cytokines including TNF-α, IL-6, and IL-1β from asthma sputum cells was abrogated by IL-37. Therefore, IL-37 is important in modulating asthma by suppressing production of pro-inflammatory cytokines (31).

Together, the expression and activities of IL-37 are important in the maintenance of immune homeostasis. It is therefore hypothesized that IL-37 is a natural inhibitor of allergic airway inflammation *via* suppressing eosinophil activation. In an attempt to further evaluate the anti-inflammatory mechanism of IL-37 in allergic asthma, *in vitro* intracellular signaling mechanisms of TLR2-activated human eosinophils and the *in vivo* immunoregulatory activities of IL-37 in house dust mite (HDM)-humanized allergic asthmatic mice, ovalbumin (OVA)/HDM-induced allergic asthmatic mice have been investigated. Among five different IL-37 splice variants, IL-37b is the largest and best characterized variant (32). This study was therefore performed using recombinant human IL-37b.

## Materials and Methods

### Reagents

Recombinant human IL-37b protein was purchased from R&D Systems, Inc., Minneapolis, MN, USA. PGN was bought from Invivogen Inc., San Diego, CA, USA. Albumin from chicken egg white (OVA) was purchased from Sigma-Aldrich Corp., St. Louis, MO, USA. HDM (*Dermatophagoides pteronyssinus*) was obtained from Greerlabs Inc., Lenoir, NC, USA.

### Blood Buffy Coat

Fresh human buffy coat was obtained from healthy volunteers of Hong Kong Red Cross Blood Transfusion Service for the purification of primary human eosinophils. The experimental procedure using human eosinophils purified from buffy coats was approved by Clinical Research Ethics Committee, The Chinese University of Hong Kong-New Territories East Cluster Hospitals, according to the 1964 Declaration of Helsinki and its later amendments and informed written consent was obtained from all subjects.

### Mice

Inbred male BALB/c mice (6–8 weeks old) and female non-obese diabetic/severe combined immunodeficiency (NOD/SCID) (5–6 weeks old) were bred under specific pathogen-free conditions and kept at Laboratory Animal Services Center, The Chinese University of Hong Kong. All animal experimentations were performed in accordance with the principles outlined in the Animal Experimentation Ethics Committee (AEEC) guide for the Care and Use of Laboratory Animals, with the approval of the AEEC of The Chinese University of Hong Kong.

### Patients

Allergic asthmatic patients aged 30–40 years with HDM sensitization (*n* = 4) and sex- and age-matched non-atopic healthy control subjects (*n* = 4) were recruited from The Prince of Wales Hospital, Hong Kong. EDTA anticoagulated blood (50 ml) obtained from each subject was diluted (1:1 v/v) with cold phosphate-buffered saline (PBS) and PBMC were then harvested by using Ficoll density gradient centrifugation (GE Healthcare Life Sciences, Piscataway, NJ, USA). Purified PBMC (2 × 10^7^) were used for intraperitoneal injection (i.p.) into SCID mouse to construct humanized mice. All recruited subjects were ethnic Chinese. The above clinical protocol were approved by the Clinical Research Ethics Committee, The Chinese University of Hong Kong-New Territories East Cluster Hospitals, and informed written consent was signed by all subjects or their parents in accordance with the 1964 Declaration of Helsinki and its later amendments.

### Purification of Eosinophils From Human Buffy Coat

Phosphate-buffered saline diluted fresh human blood buffy coat was centrifuged using the 1.082 g/ml isotonic Percoll solution (GE Healthcare Life Sciences) for 20 min at 900 *g*. After RBC lysis, the obtained granulocyte fraction was collected to isolate eosinophils by anti-CD16 antibody-coated magnetic beads (Miltenyi Biotec, Bergisch Gladbach, Germany), followed by the depletion of CD16-positive cells by loading the cells onto a LS + column (Miltenyi Biotec) within a magnetic field. The drop-through fraction containing eosinophils with purity of at least 99% assessed by Hemacolor rapid blood smear stain (E Merck Diagnostica, Darmstadt, Germany) were collected (33).

### Coculture of Human Eosinophils With Human Bronchial Epithelial BEAS-2B Cells

The human bronchial epithelial cell line BEAS-2B cells (American Type Culture Collections, Manassas, VA, USA) were maintained in LHC-8 medium (Thermo Fisher Scientific, Rockford, IL, USA). For the experiment of coculture, the medium was changed to RPMI 1640 supplemented with 10% fetal bovine serum (FBS, Gibco Invitrogen Corp., Carlsbad, CA, USA). Eosinophils (3 × 10^5^) and BEAS-2B cells (1 × 10^5^) were cocultured with or without IL-37b (100 or 200 ng/ml) pre-treatment for 10 min, followed by PGN (10 µg/ml) stimulation for further 20 h.

### Flow Cytometric Analysis of Intracellular Signaling Molecules

Cocultured cells were pre-treated with IL-37b (200 ng/ml) for 10 min and stimulated with TLR2 ligand PGN (10 µg/ml) for 20 min. BEAS-2B cells and eosinophils were collected and fixed with fixation buffer (BioLegend Inc., San Diego, CA, USA) for 30 min at room temperature, followed with Intracellular Staining Permeabilization Wash Buffer (BD Biosciences, San Jose, CA, USA) at 4°C for 30 min. Cells were then stained with fluorescence dye-conjugated antibodies of mouse anti-human phosphorylated IκBα, Akt, extracellular signal-regulated kinase (ERK)1/2, or corresponding isotypic control antibody (BD Biosciences). After washing, cells were analyzed with FACSCalibur flow cytometer (BD Biosciences).

### Identification and Transcriptional Profile of IL-37b Target Genes

RNA isolation, library preparation for transcriptome sequencing and clustering and sequencing were performed by Novogene Co., Ltd., Beijing, China. Briefly, BEAS-2B cells and eosinophils were cocultured with different treatments, including IL-37b (200 ng/ml), PGN (10 µg/ml), PGN (10 µg/ml) plus IL-37b (200 ng/ml), with untreated cocultured cells as normal control, for 20 h before harvest. Total RNA was extracted from cocultured eosinophils using QIAzol reagent (Qiagen, Valencia, CA, USA). RNA (1 µg per sample) was applied as starting material for the RNA sample preparation. Sequencing libraries were created using NEBNext^®^ Ultra™ RNA Library Prep Kit for Illumina^®^ (NEB, USA) and index codes were used to attribute sequence to each sample. The clustering of the index-coded samples was analyzed on a cBot Cluster Generation System with TruSeq PE Cluster Kit v3-cBot-HS (Illumina, Inc., San Diego, CA, USA). After cluster generation, the library preparations were sequenced on an Illumina Hiseq 4000 platform to generate 150 bp paired-end reads. By clearing low quality reads from raw data, clean data/reads were obtained and were subsequently mapped to human genome assembly by HISAT (version 2.0.4). Fragments per kilobase of transcript per million mapped reads (FPKM) were calculated using HTSeq (version 0.6.1) to analyze gene expression levels. Differential expression analysis of four treatments was performed by the DESeq (version 1.10.1, Bioconductor). Genes with *P* < 0.05 were stated as differentially expressed. Gene ontology (GO) enrichment was assessed by DAVID Bioinformatics Resources 6.8 (National Institute of Allergy and Infectious Diseases, NIH). GO terms with Benjamini–Hochberg corrected *P* < 0.05 were defined to be significantly enriched by differentially expressed genes (DEG). RNA sequence data have been deposited at NCBI Sequence Read Archive under SRP study accession number SRP138008.

### Validation of RNA-Seq Result and mRNA Levels of Cytokines/Chemokines Expression in Cocultured Cells by Real-Time Quantitative PCR

Total RNA was reverse transcribed into first-strand complementary DNA by using PrimeScript™ RT Master Mix (Takara Bio Inc., Shiga, Japan). The expression of gene *TNF-α, IL-1β, IL-6, CCL2*, and *CXCL8* in cocultured eosinophils and BEAS-2B cells, as well as nine target genes screened from RNA-seq result were quantified using SYBR^®^ Premix Ex Taq™ (Takara Bio Inc., Shiga, Japan), with corresponding primers (Table S1 in Supplementary Material). The GAPDH housekeeping gene was used as an internal reference and 2^−ΔCt (Ct, target gene-Ct, GAPDH)^ was used to calculate the relative gene expression.

### Humanized SCID Mice With Allergic Asthma

Humanized asthmatic NOD/SCID (non-obese diabetic/severe combined immunodeficiency) mice were developed as our previously described (34). PBMCs (2 × 10^7^) from allergic asthmatic patients with HDM sensitization or sex- and age-matched healthy control subjects were i.p. transplanted into NOD/SCID mice on day 1. HDM (50 µg) was intratracheally instilled on days 2, 4, and 8. Recombinant human IL-37b (1 µg) or 200 μl PBS was i.v. injected on days 1, 2, 4, 8, 12, 16, and 19. The humanized SCID mice were sacrificed for analysis on day 20.

### Quantification of Cytokine and Chemokine Concentration

The concentrations of human CCL2 and CXCL8 in culture supernatant were analyzed using ELISA kit (BioLegend). Human TNF-α, IL-1β, and IL-6 and murine IL-4, IL-5, IL-6, IL-13, IL-17, CCL5, and CCL11 in plasma or lung homogenate were quantified with Human or Mouse Cytokine Milliplex MAP assay kit (Millipore Corporation, Billerica MA, USA) on Bio-Plex 200 system (Bio-Rad Laboratories, Hercules, CA, USA).

### Histological Examination

Hematoxylin and eosin (H&E) staining and periodic acid-Schiff (PAS) staining were performed as previously reported (35). Briefly, lung tissues were obtained, fixed with 4% paraformaldehyde, and embedded in paraffin. Sections (5 µm) were stained with H&E staining kit (Beyotime Inc., Jiangsu, China) for assessing the general morphology and inflammatory cells infiltration. For the detection of goblet cells in the bronchia, the lung sections were stained with PAS staining kit (Sigma-Aldrich) followed with hematoxylin staining (Beyotime). After H&E and PAS staining, lung sections were dehydrated, mounted, and examined under Leica DM6000 B microscope (Leica Microsystems Inc., Buffalo Grove, IL, USA).

### Statistical Analysis

All data were analyzed on Statistical Package for the Social Sciences statistical software for Mac OS, version 22. Differences between groups were evaluated by one-way ANOVA. Data were presented as mean ± SD. Differences with *P* < 0.05 were considered statistically significant.

## Results

### Effect of IL-37b on Cytokines/Chemokines Release in the Eosinophils-BEAS-2B Cells Coculture Upon Stimulation With TLR2 Ligand PGN

For the *in vitro* study of suppressing allergic inflammation, eosinophils and BEAS-2B cells were cocultured to mimic the microenvironment in the asthmatic lung tissue in which bronchial epithelial cells interact with eosinophils (36). Since IL-18Rα and IL-1R8 are receptors for mediating the anti-inflammatory activities of IL-37b (24, 29), both IL-18Rα and IL-1R8 were detected on the surface of eosinophils and BEAS-2B cells by flow cytometry (Figures S1A,B in Supplementary Material). MTT assay and 7-AAD viability staining assay were performed on BEAS-2B cells and eosinophils, respectively, to delineate the non-cytotoxic dosage range of IL-37b for the subsequent *in vitro* study. Both assays demonstrated that IL-37b (10–1,000 ng/ml) did not produce any remarkable effect (*P* > 0.05) on both cell proliferation and viability (Figures S2A,B in Supplementary Material).

**Figure 1:**
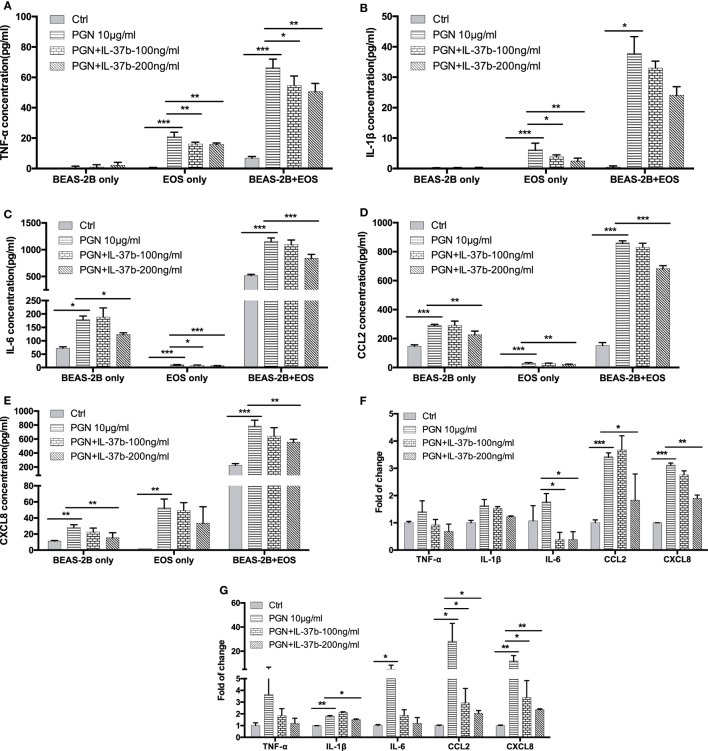
Effect of IL-37b on cytokine/chemokine release in human eosinophil-BEAS-2B coculture upon stimulation by TLR2 ligand PGN. Human eosinophils (3 × 10^5^) and BEAS-2B cells (1 × 10^5^) were cultured either together or separately with or without IL-37b (100 and 200 ng/ml) pre-treatment for 10 min, followed by stimulation with PGN (10 µg/ml) for 20 h. Release of TNF-α **(A)**, IL-1β **(B)**, IL-6 **(C)**, CCL2 **(D)**, and CXCL8 **(E)** were determined. The mRNA expression of gene *TNF-α, IL-1β, IL-6, CCL2*, and *CXCL8* in cocultured eosinophils **(F)** and BEAS-2B **(G)** were analyzed by qPCR. Abbreviations: EOS, eosinophils; TLR2, toll-like receptor-2; PGN, peptidoglycan; TNF, tumor necrosis factor. Results are shown as mean ± SD of triplicate experiments. *P < 0.05, **P < 0.01, and ***P < 0.001 when compared between the denoted groups.

Our previous studies have shown the production of cytokines and chemokines in the coculture of eosinophils and BEAS-2B cells significantly increased when compared with culture of eosinophils or BEAS-2B cells alone, and their expression could be further upregulated upon the stimulation by NOD2 ligand MDP (13, 37, 38). In this *in vitro* study, bacterial TLR2 ligand PGN was used as the stimulus because TLR2 recognizes the bacterial cell wall components to initiate the subsequent innate immune response and antigen-specific Th2 immunity in allergic asthma (39, 40). As shown in Figure 1, PGN (10 µg/ml) significantly increased inflammatory cytokines TNF-α, IL-1β, and IL-6 production and chemokines CCL2 and CXCL-8 release in eosinophils or BEAS-2B cells alone as well as coculture, except for nearly undetectable TNF-α and IL-1β production in BEAS-2Bs alone. The PGN-induced TNF-α release in eosinophils alone and eosinophils-BEAS-2B cells coculture were downregulated by IL-37b (100 and 200 ng/ml, all *P* < 0.05, Figure 1A). Moreover, the PGN-induced IL-1β release from eosinophils alone but not eosinophils-BEAS-2B cells coculture could also be significantly suppressed by IL-37b (*P* < 0.05, Figure 1B). The *in vitro* upregulated production of inflammatory cytokine IL-6 and chemokines CCL2 and CXCL-8 could also be notably suppressed by IL-37b (200 ng/ml, all *P* < 0.05, Figures 1C–E). The gene expression of corresponding cytokines and chemokines showed similar results with protein levels (Figures 1F,G).

### IL-37b Inhibits PGN-Induced Cytokines and Chemokines *via* the Downregulation of Intracellular Nuclear Factor (NF)-κB, PI3K–Akt, and ERK1/2 Pathways

**Figure 2:**
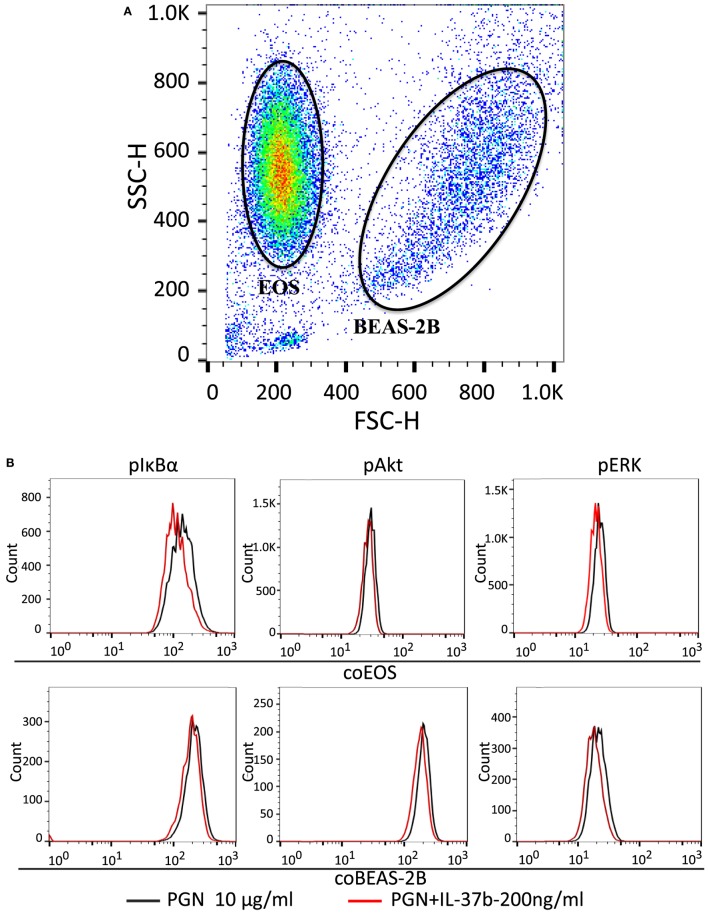

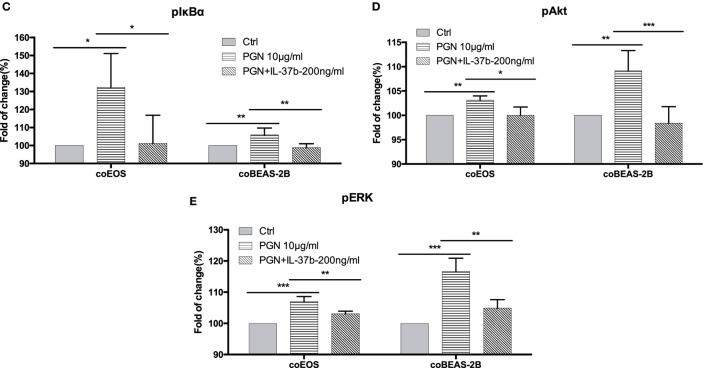
Inhibition of PGN-induced IκBα, PI3K–Akt, and ERK1/2 activation in eosinophil-BEAS-2B coculture under IL-37b treatment. Human eosinophils (1.2 × 10^6^) and BEAS-2B cells (4 × 10^5^) were cocultured with or without IL-37b (200 ng/ml) pre-treatment for 10 min followed by stimulation with PGN (10 µg/ml) for 20 min prior to fixation and permeabilization. **(A)** Eosinophils and BEAS-2B cells could be distinguished through FSC and SSC by flow cytometry. **(B)** Representative flow cytometric histograms from triplicate experiments of phosphorylated signaling molecules in cocultured eosinophils and BEAS-2B cells. Intracellular levels of phosphorylated IκBα **(C)**, phosphorylated Akt **(D)**, and phosphorylated ERK1/2 **(E)** in cells were measured by intracellular staining with specific antibodies and analyzed using flow cytometry. Results are expressed as fold of change (%) comparing with corresponding control groups and shown as mean ± SD, *n* = 3–6 per group. *P < 0.05, **P < 0.01, and ***P < 0.001 when compared between the denoted groups. Abbreviations: FSC, forward scatter; SSC, side scatter; EOS, eosinophils; coEOS, eosinophils in coculture; coBEAS-2B, BEAS-2B cells in coculture; ERK, extracellular signal-regulated kinase; PGN, peptidoglycan.

We have previously shown that bacterial PGN and TLR2 ligation could activate eosinophils by eliciting the activation of well-characterized downstream signaling mechanisms including NF-κB,PI3K–Akt, and ERK1/2 pathway, thereby accounting for the production of the inflammatory cytokines and chemokines within 30 min (41). The expressions of signaling molecules were analyzed by flow cytometry. The mixed eosinophils and BEAS-2B cells could be distinguished and analyzed separately by forward scatter and side scatter (Figure 2A). Figure 2 illustrates the underlying intracellular mechanism of immunosuppressive IL-37b in which upregulated levels of phosphorylated IκBα, Akt, and ERK1/2 in response to PGN were all markedly suppressed by IL-37b in both BEAS-2Bs and eosinophils in coculture (all *P* < 0.05, Figures 2B–E).

### Identification of IL-37b Target Genes Through Transcriptional Profiling

**Figure 3:**
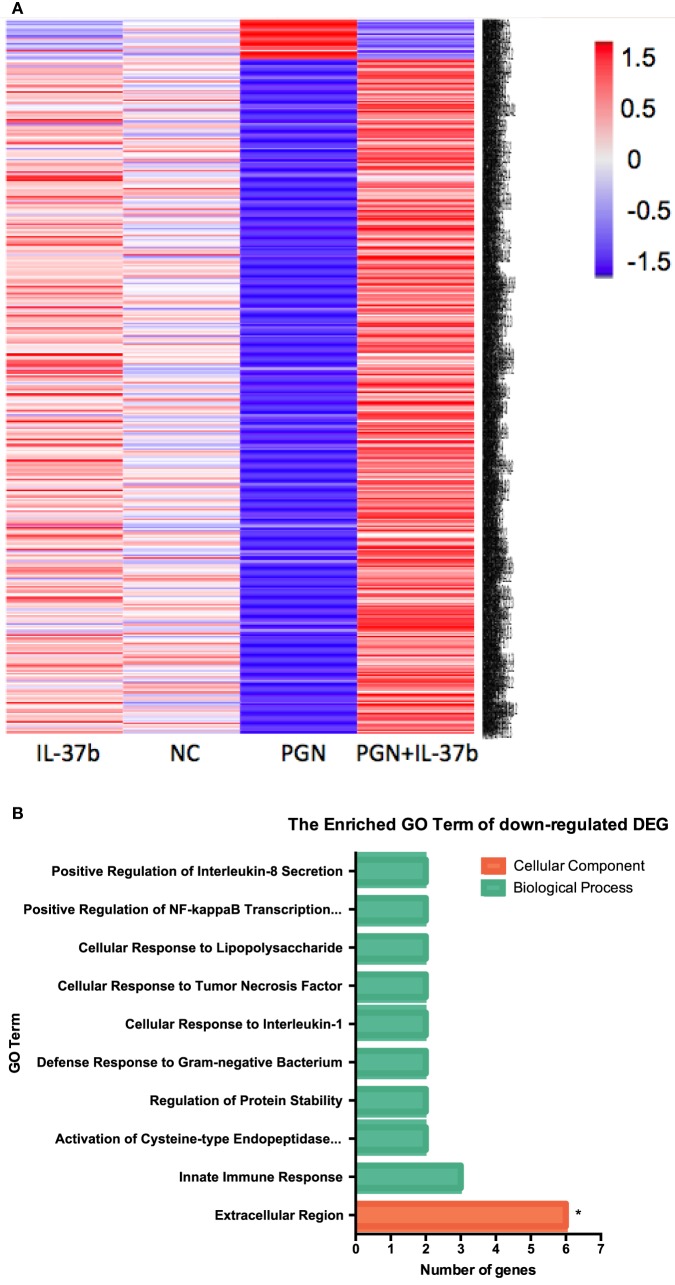

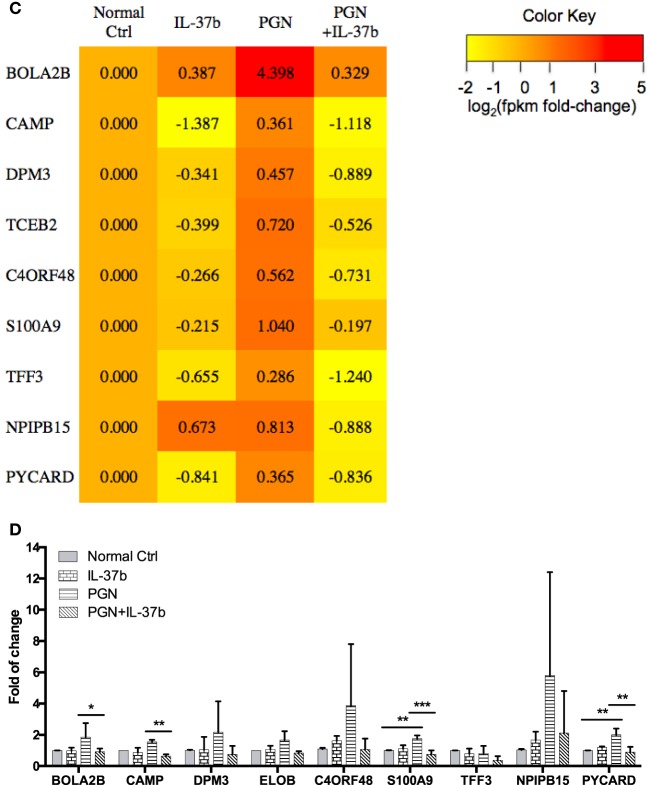
Identification of IL-37b target genes through transcriptional profiling. Human eosinophils and BEAS-2B cells were cocultured with or without IL-37b (200 ng/ml) pre-treatment for 10 min followed by stimulation with PGN (10 µg/ml) for 20 h. Total RNA of eosinophils was extracted and transcriptome analysis was performed to identify IL-37b target genes. **(A)** Heatmap of DEG between IL-37b-treated PGN sample and untreated PGN sample is shown. **(B)** Gene ontology (GO) term enrichment analysis of downregulated DEG, *P < 0.05 indicates significantly enriched GO term after Benjamini–Hochberg correction. **(C)** Fold of change for downregulated DE genes was depicted on a log_2_ scale heatmap, with a yellow-to-red gradient indicating downregulation to upregulation. **(D)** Real-time qPCR validation of RNA-seq result. The GAPDH housekeeping gene was used as the internal reference and the relative gene expression was calculated using 2^−ΔCt (Ct, target gene-Ct, GAPDH)^. Results are shown as mean ± SD, *n* = 3 per group, *P < 0.05 when compared between the denoted groups. Abbreviations: DEG, differentially expressed genes; PGN, peptidoglycan.

**Figure 4:**
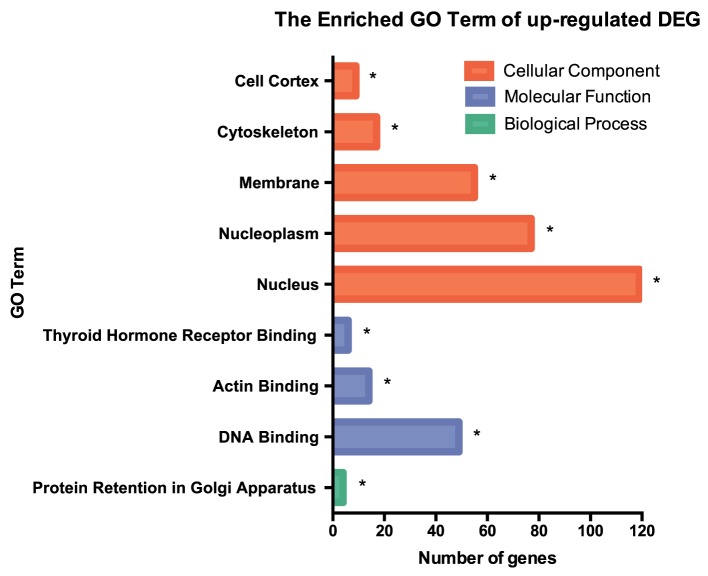
GO term enrichment analysis of upregulated DEG. Upregulated DEG for IL-37b treated PGN sample versus untreated PGN sample were sorted out with the criteria of log2 (fold-change) > 0 and *P* < 0.05, and then proceeded to GO enrichment analysis. Total 408 genes were categorized into 171 functional groups, in which 9 groups were significantly overrepresented with Benjamini–Hochberg corrected *P* < 0.05. Abbreviations: DEG, differentially expressed gene; GO, gene ontology; PGN, peptidoglycan.

RNA-seq data were collected from the analysis of eosinophils cocultured with BEAS-2B cells after different treatments. The suspended cocultured eosinophils were separated from adhesive BEAS-2B cells. According to hemacolor rapid blood smear staining, the purity of eosinophils was more than 99% (Figure S3 in Supplementary Material). DEG were screened from IL-37b treated PGN sample versus untreated PGN sample. Heatmap analysis is shown in Figure 3A. According to the criteria of log_2_ (fold-change) < 0 and *P* < 0.05, 25 downregulated DEG were identified. Excluding pseudogenes and uncategorized genes, the functions of protein coding genes *BOLA2B, CAMP, DPM3, ELOB, C4ORF48, S100A9, TFF3, NPIPB15*, and *PYCARD* were annotated (Table 1). GO enrichment analysis was performed for the genes mentioned above. Only extracellular region, a cellular component GO term, was significantly overrepresented with Benjamini–Hochberg corrected *P* < 0.05. However, we found that all other GO terms with *P* < 0.05 belonged to biological process GO term, and were mainly related to innate immune response, cytokines secretion, and signal transcription activation which may give an elucidation of the mechanism of IL-37b on eosinophils (Figure 3B; Table 2). Heatmap of the downregulated DEG was constructed using log_2_ (fpkm fold-change) to present gene expression level. As shown in Figure 3C, the upregulated expression of target genes by PGN was suppressed upon IL-37b treatment. Target genes were further validated by real-time qPCR in three biological replicates and the result was coincident with RNA-seq (Figure 3D). Also, the expression differences of *BOLA2B, CAMP, S100A9*, and *PYCARD* between PGN and PGN + IL-37b were statistically significant (Figure 3D). Upregulated DEG of IL-37b treated PGN sample versus untreated PGN sample were sorted out with the criteria of log_2_ (fold-change) > 0 and *P* < 0.05, and then proceeded to GO enrichment analysis. In total, 408 genes were categorized into 171 functional groups, in which 9 groups were significantly overrepresented with Benjamini–Hochberg corrected (*P* < 0.05) and mainly belonged to molecular function and cellular component GO terms. However, no inflammation or immune-related terms were found in upregulated DEG (Figure 4).

**Table 1:** The summary of downregulated DE genes for IL-37b-treated peptidoglycan (PGN) sample versus untreated PGN sample.

Gene symbol	Description	baseMeanA	baseMeanB	log_2_ (fold-change)	*P*-value
				B/A	
*BOLA2B*	BolA family member 2B	916.763	51.049	−4.167	0.000
*CAMP*	Cathelicidin antimicrobial peptide	372.817	124.678	−1.580	0.025
*DPM3*	Dolichyl-phosphate mannosyltransferase subunit 3	737.485	271.935	−1.439	0.030
*ELOB*	Elongin B	10,120.043	3,995.581	−1.341	0.032
*C4ORF48*	Chromosome 4 open reading frame 48	1,111.320	425.083	−1.386	0.033
*S100A9*	S100 calcium binding protein A9	14,845.446	5,897.163	−1.332	0.033
*TFF3*	Trefoil factor 3	190.483	61.848	−1.623	0.037
*NPIPB15*	Nuclear pore complex interacting protein family member B15	116.123	33.378	−1.799	0.040
*PYCARD*	PYD and CARD domain containing	1,008.439	410.357	−1.297	0.046

*Total RNA of eosinophils was extracted and transcriptome analysis was performed to identify IL-37b target genes. The downregulated DE genes (*P* < 0.05) for IL-37b treated PGN sample versus untreated PGN sample were ranked by *P*-value. A and B indicate untreated PGN and IL-37b-treated PGN, respectively*.

**Table 2:** GO analysis of downregulated genes.

Category	Annotation term	%	*P*-value	Genes	Benjamini–Hochberg corrected*P*-value
GOTERM_CC_DIRECT	GO:0005576~extracellular region	42.9	9.2E−04	*C4ORF48, CAMP, S100A9, PYCARD, TFF3, NPIPB15*	0.033
GOTERM_BP_DIRECT	GO:2000484~positive regulation of interleukin-8 secretion	14.3	4.6E−03	*CAMP, PYCARD*	0.397
GOTERM_BP_DIRECT	GO:0045087~innate immune response	21.4	9.2E−03	*CAMP, S100A9, PYCARD*	0.395
GOTERM_BP_DIRECT	GO:0050829~defense response to Gram-negative bacterium	14.3	1.9E−02	*CAMP, PYCARD*	0.511
GOTERM_BP_DIRECT	GO:0031647~regulation of protein stability	14.3	2.5E−02	*PYCARD, DPM3*	0.495
GOTERM_BP_DIRECT	GO:0071347~cellular response to interleukin-1	14.3	2.5E−02	*CAMP, PYCARD*	0.426
GOTERM_BP_DIRECT	GO:0006919~activation of cysteine-type endopeptidase activity involved in apoptotic process	14.3	2.9E−02	*S100A9, PYCARD*	0.417
GOTERM_BP_DIRECT	GO:0071356~cellular response to tumor necrosis factor	14.3	3.9E−02	*CAMP, PYCARD*	0.459
GOTERM_BP_DIRECT	GO:0071222~cellular response to lipopolysaccharide	14.3	4.0E−02	*CAMP, PYCARD*	0.424
GOTERM_BP_DIRECT	GO:0051092~positive regulation of NF-κB transcription factor activity	14.3	4.7E−02	*S100A9, PYCARD*	0.439
GOTERM_BP_DIRECT	GO:0042742~defense response to bacterium	14.3	5.1E−02	*CAMP, S100A9*	0.433
GOTERM_CC_DIRECT	GO:0070062~extracellular exosome	35.7	5.5E−02	*CAMP, S100A9, ELOB, BOLA2B, TFF3*	0.636
GOTERM_MF_DIRECT	GO:0005515~protein binding	35.7	7.3E−02	*S100A9, TCEB2, PYCARD, TFF3, DPM3*	0.826

*GO enrichment analysis was assessed for genes in Table 1 by DAVID Bioinformatics Resources 6.8 (National Institute of Allergy and Infectious Diseases, NIH). GO terms with Benjamini–Hochberg corrected *P* < 0.05 were considered significantly enriched by DEG*.

*CC, cellular component; BP, biological process; MF, molecular function; GO, gene ontology; NF, nuclear factor; DEG, differentially expressed genes*.

### The Anti-Inflammatory Activity of IL-37b on Humanized Asthmatic Mice

**Figure 5:**
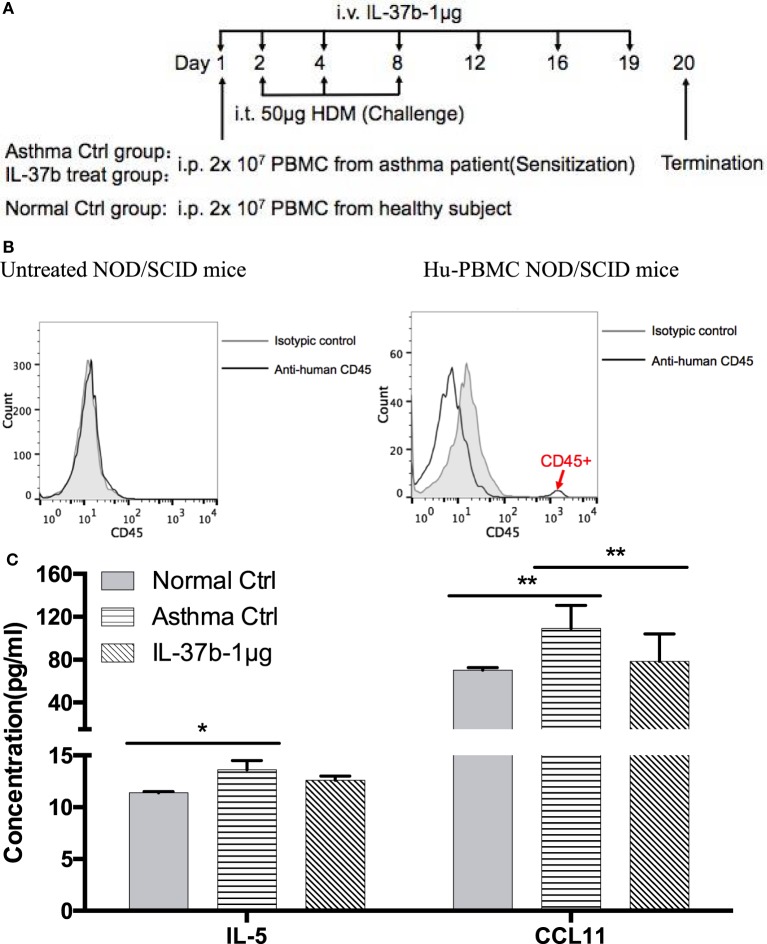

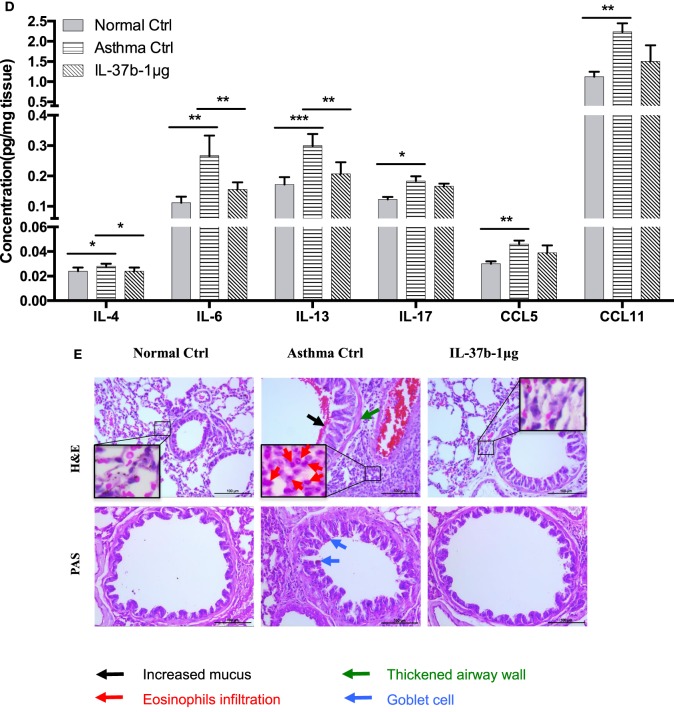
*In vivo* effects of human recombinant IL-37b on the development of asthmatic features in humanized allergic asthmatic mice. **(A)** Timeline protocol of asthmatic human-PBMC NOD/SCID mice and IL-37b administration. **(B)** Human CD45^+^ cells were assessed just before termination on day 19 to ensure human PBMCs existed stably in the blood of human-PBMC NOD/SCID mice. The concentrations of **(C)** IL-5 and CCL11 in plasma, and **(D)** protein expression of IL-4, IL-6, IL-13, IL-17, CCL5, and CCL11 in lung homogenate were analyzed. **(E)** Representative H&E and PAS staining of lung sections (200× magnification) with essentially similar results are shown. The black, red, green, and blue arrows denote increased mucus, eosinophils infiltration, thickened airway wall, and goblet cells, respectively. Results are shown as mean ± SD, *n* = 4 per group. *P < 0.05, **P < 0.01, and ***P < 0.001 when compared between the denoted groups. Data are representative from two independent experiments with essentially similar results. Abbreviations: PBMCs, peripheral blood mononuclear cells; NOD/SCID, non-obese diabetic/severe combined immunodeficiency; PAS, periodic acid-Schiff; H&E, hematoxylin and eosin.

Humanized allergic asthmatic NOD/SCID mice were applied as preclinical murine model to further evaluate the therapeutic potential of IL-37b for human allergic asthma (Figure 5A). Human CD45^+^ cells were assessed just before termination to ensure human PBMCs existed stably in the blood of human PBMC NOD/SCID mice (Figure 5B).

IL-37b treated group resulted in a remission of the airway inflammation. The levels of eosinophil activators CCL11 and IL-5 were significantly increased in plasma of humanized asthma control group, and CCL11 restored to normal level upon IL-37b administration (all *P* < 0.05, Figure 5C). Moreover, IL-37b could significantly suppress the induced concentrations of Th2 and asthma-related cytokines IL-4, IL-6, and IL-13 in lung homogenate of asthmatic human PBMC NOD/SCID mice (all *P* < 0.05, Figure 5D). Reductions were also observed on IL-17, CCL5, and CCL11 upon the treatment of IL-37b (Figure 5D). Similarly, histopathological results of lung tissue illustrated that IL-37b could mitigate the enhanced mucus, eosinophil infiltration, thickened airway wall, and goblet cells in the lung tissue of humanized allergic asthmatic mice (Figure 5E). Regarding inflammatory cell infiltration of lymphocytes, macrophages, eosinophils, neutrophils, as well as total cells in BALF, cell number of macrophages, eosinophils, and total inflammatory cells was 8.80 × 10^5^, 2.41 × 10^5^, and 11.42 × 10^5^, respectively, which were significantly increased in asthmatic mice when compared with normal control mice. The cell number decreased to 7.04 × 10^5^, 1.22 × 10^5^, and 8.44 × 10^5^, respectively, after IL-37b treatment. Similar IL-37-mediated anti-allergic inflammatory activities, such as the suppression of inflammatory cytokine/chemokine expression, specific IgE, inflammatory cell infiltration, thickened airway wall, collagen deposition, mucus and lung fibrosis together with upregulated Treg in lung tissue, etc., were shown in OVA-induced allergic asthmatic mice (Figure S4 in Supplementary Material) and HDM-induced allergic asthmatic mice (Figure S5 in Supplementary Material).

## Discussion

IL-37 has been shown to play immunoregulatory roles in animal models of myocardial infarction, RA, diabetes, fungal infection, septic shock, colitis, hepatitis, contact-hypersensitivity, psoriasis, and fibrosarcoma (22, 26–30, 42–46). Protective functions have also been reported for IL-37b on allergic airway inflammation induced by OVA, probably *via* IL-18Rα and the orphan receptor IL-1R8 (29, 47). Eosinophilia is a prominent characteristic of allergic asthma and related with asthma severity. However, the underlying cellular mechanisms by which IL-37b regulates human eosinophils in allergic asthma have not been clearly investigated. In this study, we illustrated that IL-37b could suppress transcriptional levels of *PYCARD, S100A9*, and *CAMP* genes, and antagonize the activation of NF-κB, PI3K–Akt, and ERK1/2 pathways in eosinophils, which elucidate the intracellular signaling cascade to suppress the bacterial TLR2-mediated activation in eosinophils upon interaction with human bronchial epithelial cells, and ameliorate the exacerbated allergic airway inflammation in HDM-humanized asthmatic mice.

Cascade reactions amplified by Th2 cells promote the recruitment of eosinophils into the lung tissue, with subsequent mucus production, bronchial inflammation, and AHR and remodeling which are characteristics of allergic asthma (3, 48). During the development of asthma, eosinophils are accessible to human bronchial epithelial cells in bronchus, and their interaction upon the stimulation by allergen activates both cells to induce allergic inflammation. In our study, BEAS-2B cells, which share similar pattern for the expression of cell surface adhesion molecules and production of inflammatory cytokines and chemokines with primary human bronchial epithelial cells as demonstrated in our previous studies (49, 50), were used to coculture with human eosinophils followed by stimulation with TLR2 ligand PGN to mimic the microenvironment upon infection in the bronchus of allergic asthmatic patients. Results in Figure 1 showed the capability of IL-37b for relieving the activation of TLR2 pathway in both eosinophils and BEAS-2B cells, *via* specific signaling pathways and transcriptional regulation (Figures 2 and 3).

Using murine splenic macrophages and DCs of wild-type IL-37-transgenic mice and IL-37-transgenic with IL-1R8-deficient mice, a previous report has revealed the signaling pathway under LPS stimulation that IL-37b binds to its receptors IL-1R8 and IL-18Rα and exhibits anti-inflammatory properties through signaling molecules such as STAT3 and p62 (dok) to inhibit the kinases Fyn, TAK1, and the transcription factor NF-κB, as well as mitogen-activated protein kinase (MAPK) (24). This finding therefore elucidated the regulatory mechanism of IL-37b on murine splenic antigen-presenting cells in LPS-induced inflammation. Given that the mechanisms of IL-37b may be different in different species and cell types, the phosphorylation levels of cell signaling molecules IκB, ERK1/2, and Akt in human eosinophils upon coculture with BEAS-2B and stimulation by PGN were quantified using flow cytometry in our study. We illustrated the involvement of distinct NF-κB, ERK1/2, and Akt pathways in IL-37b suppressing the activation of eosinophils and BEAS-2B cells (Figure 2). With regard of the central roles of pro-inflammatory gene transcription for the NF-κB pathway (51), reductions of TNF-α, IL-1β, IL-6, CCL2, and CXCL-8 release in *in vitro* coculture by IL-37b may partly rely on the suppressed transcriptional activity of NF-κB. Activation of ERK1/2 and Akt pathways has been reported to involve in lung inflammation and alveolar remodeling (52, 53). Consequently, IL-37b-attenuated phosphorylation of ERK1/2 and Akt in eosinophils and BEAS-2B cells, at least in part, can account for the diminished airway thickness in asthmatic mice.

**Figure 6:**
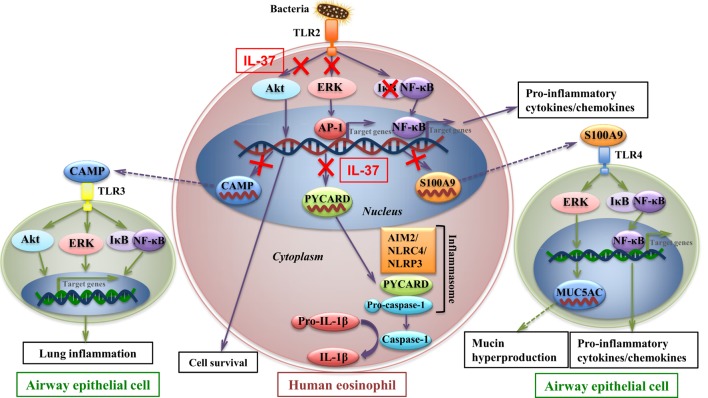
IL-37-mediated intracellular signaling cascades in eosinophils for the suppression of allergic airway inflammation. Interaction of toll-like receptor-2 (TLR2) with bacterial cell wall component leads to the activation of intracellular signaling cascades in eosinophils with subsequent intercellular interaction with airway epithelial cells. Signaling molecules Akt, extracellular signal-regulated kinase (ERK)1/2, and IκB are phosphorylated to initiate the downstream transcription of target genes for cell survival and pro-inflammatory cytokines and chemokines which deteriorate allergic inflammation. Furthermore, transcriptional level of *CAMP, PYCARD*, and *S100A9* is upregulated in allergic inflammation. CAMP (LL37) protein is capable of triggering TLR3 on bronchial epithelial cells, which also activates signaling molecules and results in asthma deterioration. PYCARD functions as an adapter in diverse types of inflammasomes, such as AIM2 inflammasome, NLRC4 inflammasome, NLRP3 inflammasome, to activate caspase-1 and lead to the maturation of pro-inflammatory cytokine such as IL-1β. S100A9 triggers the production of mucin protein MUC5AC in airway epithelial cells, which contributes to mucin hyper-production in airway, and activates nuclear factor-κB pathway to induce pro-inflammatory cytokines and chemokines, which result in exacerbation of airway inflammation. IL-37 is capable of attenuating phosphorylation of Akt, ERK1/2, and IκB in eosinophils, with subsequent downregulating transcriptional level of *CAMP, PYCARD*, and *S100A9* in eosinophils, thereby ameliorating allergic airway inflammation.

To further investigate the molecular mechanisms mediated by IL-37b, RNA-sequencing, bioinformatics, and real-time qPCR validation were performed on cocultured eosinophils. Genes *PYCARD* (apoptosis-associated speck-like protein containing a CARD), *S100A9*, and *CAMP* (cathelicidin antimicrobial peptide LL-37) were suggested to play pivotal roles in IL-37b anti-allergic inflammatory activities. PYCARD is well known for its function as an adapter in diverse types of inflammasomes to activate caspase-1 and lead to the maturation of pro-inflammatory cytokines (54). PYCARD also acts on inflammasome independently with subsequent regulation on MAPK activity, NF-κB activation, and cytokines/chemokines expression (55, 56). S100A9 protein plays a vital role in inflammation and immune response (57–59). The concentration of S100A9 was elevated in BALF of asthmatic patients and correlated with plasma IgE concentration (60, 61). It has been shown that S100A9 triggers the production of mucin protein MUC5AC to contribute to mucin hyper-production in airway and activates NF-κB pathway to induce pro-inflammatory cytokines and chemokines (62). CAMP is the unique cathelicidin-family antimicrobial peptide LL-37 found in human (63–65). Our recent study has demonstrated that LL-37 promotes asthma deterioration (33). Furthermore, *PYCARD, S100A9*, and *CAMP* are annotated to inflammation and immune-related GO terms, which further illustrate their contributions in inflammation and immune response (all *P* < 0.05, Table 2; Figure 3B). Therefore, downregulated transcriptional level of these genes, at least partly, is responsible for the amelioration of allergic airway inflammation in IL-37b-treated mice. The IL-37-mediated intracellular signaling cascades in eosinophils for the suppression of allergic inflammation are illustrated in Figure 6.

Adopting our established humanized murine mice (34), chimeric allergic asthmatic mice with humanized peripheral blood cells were used to mimic human allergic asthma. Results from the humanized mice provide more clinical relevant biochemical basis for future therapeutic application of IL-37b on the treatment of allergic asthma. In OVA-induced allergic airway inflammation mice (Figure S4 in Supplementary Material) and HDM-induced allergic asthmatic mice (Figure S5 in Supplementary Material), amelioration on asthma exacerbation was indicated in IL-37b-treated groups, which is in concordance with the result of previous publications (66, 67). Noteworthy, IL-37b administrated at challenge stage showed higher efficacy on ameliorating asthma exacerbation when compared with that of sensitization stage. This finding indicates that IL-37b may be beneficial for therapeutic treatment, but less effective in prophylactic treatment. Nevertheless, further studies are needed to estimate the feasibility of IL-37b for clinical application, including efficacy and safety, and more in-depth mechanistic study is on-going.

A recently study reported the inhibition of IL-37b on HDM-induced pulmonary eosinophilia and AHR, without affecting Th2-related cytokine production (67). This discrepancy comparing with the suppression of local and systemic Th2 cytokines in our present study (Figures S4 and S5 in Supplementary Material) may be due to different administration route and dosage. In Lv et al. study, mice was intranasally (i.n.) administrated with IL-37b (200 ng). Nasal mucosa is a barrier for protein that can decrease the nasal bioavailability of high molecular weight protein ((1,000 Da) (68, 69). Therefore, most intranasal administrated IL-37b (MW = 19.4 kDa) may enter respiratory tract to interact with local pro-inflammatory cells and mediators. Together with the mucociliary clearance and enzymatic degradation in nasal mucosa (70, 71), the bioavailability of intranasal IL-37b can be further decreased, resulting in short half-life. Similar decreasing activity of i.n. administrated IL-37b (500 ng) was also observed in our study (data not shown). Therefore, our present i.v. systemic administration of IL-37b not only benefits local pulmonary inflammation but also downregulates inflammatory cells migration from circulation into pulmonary vasculature and airway submucosa, together with suppression of Th2 and inflammatory cytokine and chemokine production.

In conclusion, our study has demonstrated novel intracellular mechanism of IL-37b on human eosinophil axis by which IL-37b can suppress bacterial TLR2-mediated activation in eosinophils upon interacting with human bronchial epithelial cells, thereby contributing to the remission of allergic airway inflammation. Together with the result of preclinical studies using humanized mice, IL-37b may be used as a novel treatment modality for allergic asthmatic patients, with least side effects comparing with conventional steroid treatment. Given the pivotal role of eosinophils in many other allergic diseases such as atopic dermatitis, IL-37b may also be a potential therapeutic agent for various eosinophilic disorders.

## Ethics Statement

The experimental procedure using human eosinophils purified from buffy coats was approved by Clinical Research Ethics Committee of The Chinese University of Hong Kong-New Territories East Cluster Hospitals according to the 1964 Declaration of Helsinki and its later amendments and informed written consent was obtained from all subjects. All recruited subjects were ethnic Chinese. The above protocol were approved by the Clinical Research Ethics Committee of The Chinese University of Hong Kong-New Territories East Cluster Hospitals, and informed written consent was obtained from all subjects or their parents in accordance with the 1964 Declaration of Helsinki and its later amendments. All animal experimentations were conducted in accordance with the principles outlined in the Animal Experimentation Ethics Committee (AEEC) guide for the Care and Use of Laboratory Animals, as approved by the AEEC of The Chinese University of Hong Kong.

## Author Contributions

JZ, JD, PJ, TL, LN, DJ, CL, and C-KW contributed to the conception and design of the study. JZ, JD, LJ, DL, MT, and DJ performed experiment and analyzed data. JZ, CL, and C-KW drafted and revised the manuscript.

## Conflict of Interest Statement

The authors declare that the research was conducted in the absence of any commercial or financial relationships that could be construed as a potential conflict of interest.

## References

[1] GBD 2015 Chronic Respiratory Disease Collaborators. Global, regional, and national deaths, prevalence, disability-adjusted life years, and years lived with disability for chronic obstructive pulmonary disease and asthma, 1990-2015: a systematic analysis for the Global Burden of Disease Study 2015. Lancet Respir Med (2017) 5(9):691–706. 10.1016/S2213-2600(17)30293-X 28822787 PMC5573769

[2] Romanet-Manent S Charpin D Magnan A Lanteaume A Vervloet D EGEA Cooperative Group. Allergic vs nonallergic asthma: what makes the difference? Allergy (2002) 57(7):607–13. 10.1034/j.1398-9995.2002.23504.x 12100301

[3] Lambrecht BN Hammad H The immunology of asthma. Nat Immunol (2015) 16(1):45–56. 10.1038/ni.3049 25521684

[4] Herrick CA Bottomly K. To respond or not to respond: T cells in allergic asthma. Nat Rev Immunol (2003) 3(5):405–12. 10.1038/nri1084 12766762

[5] Oettgen HC Geha RS. IgE regulation and roles in asthma pathogenesis. J Allergy Clin Immunol (2001) 107(3):429–40. 10.1067/mai.2001.113759 11240941

[6] Galli SJ Tsai M Piliponsky AM. The development of allergic inflammation. Nature (2008) 454(7203):445–54. 10.1038/nature07204 18650915 PMC3573758

[7] Schroder NW Crother TR Naiki Y Chen S Wong MH Yilmaz A Innate immune responses during respiratory tract infection with a bacterial pathogen induce allergic airway sensitization. J Allergy Clin Immunol (2008) 122(3):595–602.e5. 10.1016/j.jaci.2008.06.038 18774395 PMC3052793

[8] Ritchie AI Farne HA Singanayagam A Jackson DJ Mallia P Johnston SL. Pathogenesis of viral infection in exacerbations of airway disease. Ann Am Thorac Soc (2015) 12(Suppl 2):S115–32. 10.1513/AnnalsATS.201503-151AW 26595727

[9] Takeuchi O Akira S Pattern recognition receptors and inflammation. Cell (2010) 140(6):805–20. 10.1016/j.cell.2010.01.022 20303872

[10] Fulkerson PC Rothenberg ME. Targeting eosinophils in allergy, inflammation and beyond. Nat Rev Drug Discov (2013) 12(2):117–29. 10.1038/nrd3838 23334207 PMC3822762

[11] Romagnani S. The role of lymphocytes in allergic disease. J Allergy Clin Immunol (2000) 105(3):399–408. 10.1067/mai.2000.104575 10719286

[12] Murphy K Travers P Walport M Janeway C Janway’s Immunobiology. 8th ed USA: Garland Science (2012). xix, 868 p.

[13] Wong CK Wang CB Ip WK Tian YP Lam CW. Role of p38 MAPK and NF-kB for chemokine release in coculture of human eosinophils and bronchial epithelial cells. Clin Exp Immunol (2005) 139(1):90–100. 10.1111/j.1365-2249.2005.02678.x 15606618 PMC1809270

[14] Wang CB Wong CK Ip WK Li ML Tian YP Lam CW. Induction of IL-6 in co-culture of bronchial epithelial cells and eosinophils is regulated by p38 MAPK and NF-kappaB. Allergy (2005) 60(11):1378–85. 10.1111/j.1398-9995.2005.00884.x 16197469

[15] Cheung PF Wong CK Ho AW Hu S Chen DP Lam CW. Activation of human eosinophils and epidermal keratinocytes by Th2 cytokine IL-31: implication for the immunopathogenesis of atopic dermatitis. Int Immunol (2010) 22(6):453–67. 10.1093/intimm/dxq027 20410259

[16] Wong CK Hu S Leung KM Dong J He L Chu YJ NOD-like receptors mediated activation of eosinophils interacting with bronchial epithelial cells: a link between innate immunity and allergic asthma. Cell Mol Immunol (2013) 10(4):317–29. 10.1038/cmi.2012.77 23524653 PMC4003204

[17] Nold MF Nold-Petry CA Zepp JA Palmer BE Bufler P Dinarello CA. IL-37 is a fundamental inhibitor of innate immunity. Nat Immunol (2010) 11(11):1014–22. 10.1038/ni.1944 20935647 PMC3537119

[18] Kumar S Hanning CR Brigham-Burke MR Rieman DJ Lehr R Khandekar S Interleukin-1F7B (IL-1H4/IL-1F7) is processed by caspase-1 and mature IL-1F7B binds to the IL-18 receptor but does not induce IFN-gamma production. Cytokine (2002) 18(2):61–71. 10.1006/cyto.2002.0873 12096920

[19] Bufler P Gamboni-Robertson F Azam T Kim SH Dinarello CA. Interleukin-1 homologues IL-1F7b and IL-18 contain functional mRNA instability elements within the coding region responsive to lipopolysaccharide. Biochem J (2004) 381(Pt 2):503–10. 10.1042/BJ20040217 15046617 PMC1133858

[20] Shuai X Wei-min L Tong YL Dong N Sheng ZY Yao YM. Expression of IL-37 contributes to the immunosuppressive property of human CD4+CD25+ regulatory T cells. Sci Rep (2015) 5:14478. 10.1038/srep14478 26411375 PMC4585986

[21] Bufler P Azam T Gamboni-Robertson F Reznikov LL Kumar S Dinarello CA A complex of the IL-1 homologue IL-1F7b and IL-18-binding protein reduces IL-18 activity. Proc Natl Acad Sci U S A (2002) 99(21):13723–8. 10.1073/pnas.212519099 12381835 PMC129755

[22] Luo Y Cai X Liu S Wang S Nold-Petry CA Nold MF Suppression of antigen-specific adaptive immunity by IL-37 via induction of tolerogenic dendritic cells. Proc Natl Acad Sci U S A (2014) 111(42):15178–83. 10.1073/pnas.1416714111 25294929 PMC4210310

[23] Lunding L Schroder A Wegmann M Allergic airway inflammation: unravelling the relationship between IL-37, IL-18Ralpha and Tir8/SIGIRR. Expert Rev Respir Med (2015) 9(6):739–50. 10.1586/17476348.2015.1109452 26561030

[24] Nold-Petry CA Lo CY Rudloff I Elgass KD Li S Gantier MP IL-37 requires the receptors IL-18Ralpha and IL-1R8 (SIGIRR) to carry out its multifaceted anti-inflammatory program upon innate signal transduction. Nat Immunol (2015) 16(4):354–65. 10.1038/ni.3103 25729923

[25] Schröder A Lunding L Webering S Vock C Raedler D Schaub B IL-37 ameliorates experimental asthma via a mechanism that is independent from IL-18 signaling. Pneumologie (2016) 70:422 10.1055/s-0036-1572306

[26] Wu B Meng K Ji Q Cheng M Yu K Zhao X Interleukin-37 ameliorates myocardial ischaemia/reperfusion injury in mice. Clin Exp Immunol (2014) 176(3):438–51. 10.1111/cei.12284 24527881 PMC4008989

[27] Ye L Jiang B Deng J Du J Xiong W Guan Y IL-37 alleviates rheumatoid arthritis by suppressing IL-17 and IL-17-triggering cytokine production and limiting Th17 cell proliferation. J Immunol (2015) 194(11):5110–9. 10.4049/jimmunol.1401810 25917106

[28] Ballak DB van Diepen JA Moschen AR Jansen HJ Hijmans A Groenhof GJ IL-37 protects against obesity-induced inflammation and insulin resistance. Nat Commun (2014) 5:4711. 10.1038/ncomms5711 25182023

[29] Lunding L Webering S Vock C Schroder A Raedler D Schaub B IL-37 requires IL-18Ralpha and SIGIRR/IL-1R8 to diminish allergic airway inflammation in mice. Allergy (2015) 70(4):366–73. 10.1111/all.12566 25557042

[30] Moretti S Bozza S Oikonomou V Renga G Casagrande A Iannitti RG IL-37 inhibits inflammasome activation and disease severity in murine aspergillosis. PLoS Pathog (2014) 10(11):e1004462. 10.1371/journal.ppat.1004462 25375146 PMC4223056

[31] Charrad R Berraies A Hamdi B Ammar J Hamzaoui K Hamzaoui A Anti-inflammatory activity of IL-37 in asthmatic children: correlation with inflammatory cytokines TNF-alpha, IL-beta, IL-6 and IL-17A. Immunobiology (2016) 221(2):182–7. 10.1016/j.imbio.2015.09.009 26454413

[32] Boraschi D Lucchesi D Hainzl S Leitner M Maier E Mangelberger D IL-37: a new anti-inflammatory cytokine of the IL-1 family. Eur Cytokine Netw (2011) 22(3):127–47. 10.1684/ecn.2011.0288 22047735

[33] Jiao D Wong CK Tsang MS Chu IM Liu D Zhu J Activation of eosinophils interacting with bronchial epithelial cells by antimicrobial peptide LL-37: implications in allergic asthma. Sci Rep (2017) 7(1):1848. 10.1038/s41598-017-02085-5 28500314 PMC5431911

[34] Dong J Wong CK Cai Z Jiao D Chu M Lam CW. Amelioration of allergic airway inflammation in mice by regulatory IL-35 through dampening inflammatory dendritic cells. Allergy (2015) 70(8):921–32. 10.1111/all.12631 25869299

[35] Fischer AH Jacobson KA Rose J Zeller R. Hematoxylin and eosin staining of tissue and cell sections. CSH Protoc (2008) 2008:pdb.prot4986. 10.1101/pdb.prot4986 21356829

[36] Rosenberg HF Dyer KD Foster PS. Eosinophils: changing perspectives in health and disease. Nat Rev Immunol (2013) 13(1):9–22. 10.1038/nri3341 23154224 PMC4357492

[37] Wong CK Wang CB Li ML Ip WK Tian YP Lam CW. Induction of adhesion molecules upon the interaction between eosinophils and bronchial epithelial cells: involvement of p38 MAPK and NF-kappaB. Int Immunopharmacol (2006) 6(12):1859–71. 10.1016/j.intimp.2006.08.003 17052676

[38] Wong CK Dong J Lam CW Molecular mechanisms regulating the synergism between IL-32gamma and NOD for the activation of eosinophils. J Leukoc Biol (2014) 95(4):631–42. 10.1189/jlb.0813452 24295830

[39] Phipps S Lam CE Foster PS Matthaei KI. The contribution of toll-like receptors to the pathogenesis of asthma. Immunol Cell Biol (2007) 85(6):463–70. 10.1038/sj.icb.7100104 17680012

[40] Kawai T Akira S. The role of pattern-recognition receptors in innate immunity: update on toll-like receptors. Nat Immunol (2010) 11(5):373–84. 10.1038/ni.1863 20404851

[41] Wong CK Cheung PF Ip WK Lam CW. Intracellular signaling mechanisms regulating toll-like receptor-mediated activation of eosinophils. Am J Respir Cell Mol Biol (2007) 37(1):85–96. 10.1165/rcmb.2006-0457OC 17332440

[42] Kumar S McDonnell PC Lehr R Tierney L Tzimas MN Griswold DE Identification and initial characterization of four novel members of the interleukin-1 family. J Biol Chem (2000) 275(14):10308–14. 10.1074/jbc.275.14.10308 10744718

[43] Liu W Deng L Chen Y Sun C Wang J Zhou L Anti-inflammatory effect of IL-37b in children with allergic rhinitis. Mediators Inflamm (2014) 2014:746846. 10.1155/2014/746846 25177111 PMC4142748

[44] Sharma S Kulk N Nold MF Graf R Kim SH Reinhardt D The IL-1 family member 7b translocates to the nucleus and down-regulates proinflammatory cytokines. J Immunol (2008) 180(8):5477–82. 10.4049/jimmunol.180.8.5477 18390730

[45] McNamee EN Masterson JC Jedlicka P McManus M Grenz A Collins CB Interleukin 37 expression protects mice from colitis. Proc Natl Acad Sci U S A (2011) 108(40):16711–6. 10.1073/pnas.1111982108 21873195 PMC3189085

[46] Bulau AM Fink M Maucksch C Kappler R Mayr D Wagner K In vivo expression of interleukin-37 reduces local and systemic inflammation in concanavalin A-induced hepatitis. ScientificWorldJournal (2011) 11:2480–90. 10.1100/2011/968479 22235179 PMC3253525

[47] Lunding L Webering S Vock C Schroder A Raedler D Schaub B Effect of IL-37 on allergic airway inflammation. Ann American Thorac Soc (2016) 13(Suppl 1):S95–6. 10.1513/AnnalsATS.201506-380MG 27027963

[48] Kumar V Abbas AK Aster JC Robbins SL Robbins Basic Pathology. 9th ed Philadelphia, PA: Elsevier/Saunders (2013). xii, 910 p.

[49] Wong CK Cao J Yin YB Lam CW. Interleukin-17A activation on bronchial epithelium and basophils: a novel inflammatory mechanism. Eur Respir J (2010) 35(4):883–93. 10.1183/09031936.00088309 19741026

[50] Qiu HN Wong CK Chu IM Hu S Lam CW. Muramyl dipeptide mediated activation of human bronchial epithelial cells interacting with basophils: a novel mechanism of airway inflammation. Clin Exp Immunol (2013) 172(1):81–94. 10.1111/cei.12031 23480188 PMC3719934

[51] Tak PP Firestein GS NF-kappaB: a key role in inflammatory diseases. J Clin Invest (2001) 107(1):7–11. 10.1172/JCI11830 11134171 PMC198552

[52] Lee PJ Zhang X Shan P Ma B Lee CG Homer RJ ERK1/2 mitogen-activated protein kinase selectively mediates IL-13-induced lung inflammation and remodeling in vivo. J Clin Invest (2006) 116(1):163–73. 10.1172/JCI25711 16374521 PMC1319220

[53] Rao SS Mu Q Zeng Y Cai PC Liu F Yang J Calpain-activated mTORC2/Akt pathway mediates airway smooth muscle remodelling in asthma. Clin Exp Allergy (2017) 47(2):176–89. 10.1111/cea.12805 27649066

[54] Franklin BS Bossaller L De Nardo D Ratter JM Stutz A Engels G The adaptor ASC has extracellular and ‘prionoid’ activities that propagate inflammation. Nat Immunol (2014) 15(8):727–37. 10.1038/ni.2913 24952505 PMC4116676

[55] Taxman DJ Holley-Guthrie EA Huang MT Moore CB Bergstralh DT Allen IC The NLR adaptor ASC/PYCARD regulates DUSP10, mitogen-activated protein kinase (MAPK), and chemokine induction independent of the inflammasome. J Biol Chem (2011) 286(22):19605–16. 10.1074/jbc.M111.221077 21487011 PMC3103340

[56] Hasegawa M Imamura R Motani K Nishiuchi T Matsumoto N Kinoshita T Mechanism and repertoire of ASC-mediated gene expression. J Immunol (2009) 182(12):7655–62. 10.4049/jimmunol.0800448 19494289

[57] Lagasse E Clerc RG. Cloning and expression of two human genes encoding calcium-binding proteins that are regulated during myeloid differentiation. Mol Cell Biol (1988) 8(6):2402–10. 10.1128/MCB.8.6.2402 3405210 PMC363438

[58] Gebhardt C Nemeth J Angel P Hess J. S100A8 and S100A9 in inflammation and cancer. Biochem Pharmacol (2006) 72(11):1622–31. 10.1016/j.bcp.2006.05.017 16846592

[59] Nacken W Roth J Sorg C Kerkhoff C. S100A9/S100A8: myeloid representatives of the S100 protein family as prominent players in innate immunity. Microsc Res Tech (2003) 60(6):569–80. 10.1002/jemt.10299 12645005

[60] Kim DH Choi E Lee JS Lee NR Baek SY Gu A House dust mite allergen regulates constitutive apoptosis of normal and asthmatic neutrophils via toll-like receptor 4. PLoS One (2015) 10(5):e0125983. 10.1371/journal.pone.0125983 25973752 PMC4431853

[61] Sharma M Mehla K Batra J Ghosh B. Association of a chromosome 1q21 locus in close proximity to a late cornified envelope-like proline-rich 1 (LELP1) gene with total serum IgE levels. J Hum Genet (2007) 52(4):378–83. 10.1007/s10038-007-0118-5 17387579

[62] Kang JH Hwang SM Chung IY S100A8, S100A9 and S100A12 activate airway epithelial cells to produce MUC5AC via extracellular signal-regulated kinase and nuclear factor-kappaB pathways. Immunology (2015) 144(1):79–90. 10.1111/imm.12352 24975020 PMC4264912

[63] Durr UH Sudheendra US Ramamoorthy A. LL-37, the only human member of the cathelicidin family of antimicrobial peptides. Biochim Biophys Acta (2006) 1758(9):1408–25. 10.1016/j.bbamem.2006.03.030 16716248

[64] Kuroda K Okumura K Isogai H Isogai E. The human cathelicidin antimicrobial peptide LL-37 and mimics are potential anticancer drugs. Front Oncol (2015) 5:144. 10.3389/fonc.2015.00144 26175965 PMC4485164

[65] Piktel E Niemirowicz K Wnorowska U Watek M Wollny T Gluszek K The role of cathelicidin LL-37 in cancer development. Arch Immunol Ther Exp (2016) 64(1):33–46. 10.1007/s00005-015-0359-5 26395996 PMC4713713

[66] Huang N Liu K Liu J Gao X Zeng Z Zhang Y Interleukin-37 alleviates airway inflammation and remodeling in asthma via inhibiting the activation of NF-kappaB and STAT3 signalings. Int Immunopharmacol (2018) 55:198–204. 10.1016/j.intimp.2017.12.010 29268192

[67] Lv J Xiong Y Li W Cui X Cheng X Leng Q IL-37 inhibits IL-4/IL-13-induced CCL11 production and lung eosinophilia in murine allergic asthma. Allergy (2018). 10.1111/all.13395 29319845

[68] McMartin C Hutchinson LE Hyde R Peters GE. Analysis of structural requirements for the absorption of drugs and macromolecules from the nasal cavity. J Pharm Sci (1987) 76(7):535–40. 10.1002/jps.2600760709 2889824

[69] O’Hagan DT Illum L. Absorption of peptides and proteins from the respiratory tract and the potential for development of locally administered vaccine. Crit Rev Ther Drug Carrier Syst (1990) 7(1):35–97. 2257636

[70] Romeo VD deMeireles J Sileno AP Pimplaskar HK Behl CR Effects of physicochemical properties and other factors on systemic nasal drug delivery. Adv Drug Deliv Rev (1998) 29(1–2):89–116. 10.1016/S0169-409X(97)00063-X 10837582

[71] Cornaz AL Buri P Nasal-mucosa as an absorption barrier. Eur J Pharm Biopharm (1994) 40(5):261–70.

