# Supplementary material for: Anti-Allergic Inflammatory Activity of Interleukin-37 Is Mediated by Novel Signaling Cascades in Human Eosinophils
Source: Front Immunol. 2018 Jun 22;9:1445. doi: 10.3389/fimmu.2018.01445 (PMC6023969; doi:10.3389/fimmu.2018.01445)
Supplement: Supplementary file 1 [file presentation_1.PDF]

Supplementary Material

Anti-allergic inflammatory activity of interleukin-37 is mediated by novel signaling cascades in human eosinophils

Jing Zhu[1], Jie Dong[1], Lu Ji[1], Peiyong Jiang[1], Ting Fan Leung[2], Dehua Liu[3,4], Lai Guan Ng[5], Miranda Sin-Man Tsang[3,4], Delong Jiao[1], Christopher Wai-Kei Lam[6], Chun-Kwok Wong[1,3,4,*]

[1]Department of Chemical Pathology, The Chinese University of Hong Kong, Prince of Wales Hospital, Shatin, NT, Hong Kong;

[2]Department of Paediatrics, The Chinese University of Hong Kong, Prince of Wales Hospital, Shatin, NT, Hong Kong

[3]Institute of Chinese Medicine and State Key Laboratory of Phytochemistry and Plant Resources in West China, the Chinese University of Hong Kong, Hong Kong

[4]School of Chinese Medicine, The Chinese University of Hong Kong, Hong Kong;

[5]Singapore Immunology Network, Singapore;

[6]State Key Laboratory of Quality Research in Chinese Medicines, Macau Institute for Applied Research in Medicine and Health, Macau University of Science and Technology, Taipa, Macau;

***Correspondence:**

Professor Chun Kwok Wong

Department of Chemical Pathology, The Chinese University of Hong Kong, Prince of Wales Hospital, Shatin, NT, Hong Kong.

E.Mail: ck-wong@cuhk.edu.hk

Supplementary Figure S1

Figure S1A. IL-18Rα protein expression on eosinophils and BEAS-2B cells.

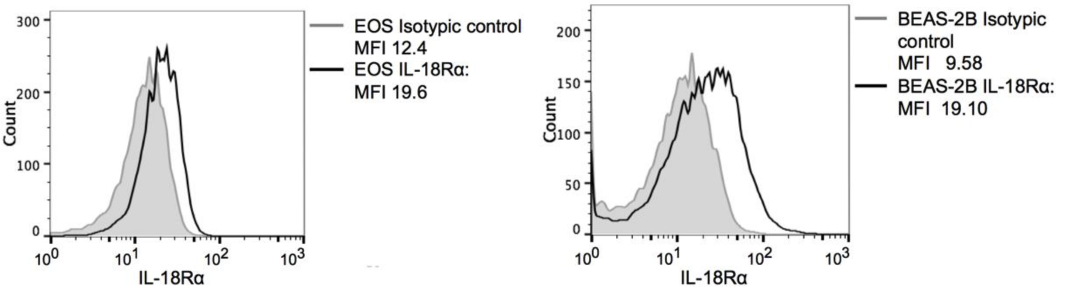

Figure S1B. IL-1R8 protein expression on eosinophils and BEAS-2B cells.

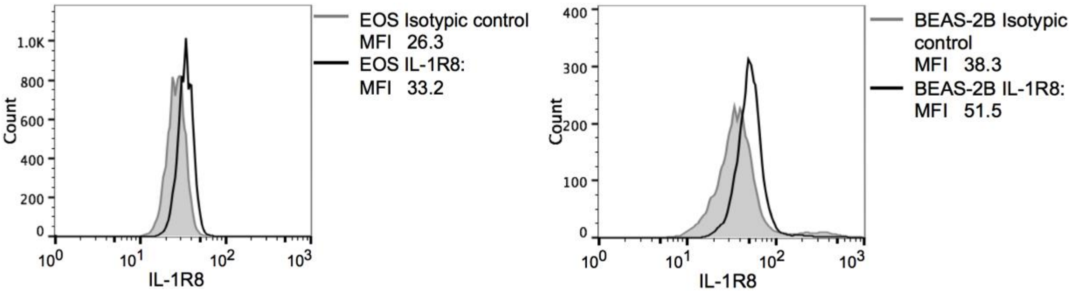

Supplementary Figure S1. The expression of IL-37b receptors on eosinophils and BEAS-2B cells.

(A) Eosinophils and BEAS-2B cells were stained with fluorescence-conjugated anti-human IL-18Rα antibody or corresponding isotypic IgG control antibody (R&D Systems Inc., Minneapolis, Minnesota, USA) and the expression of IL-18Rα were detected using flow cytometry. (B) The surface expression of IL-1R8 on eosinophils and BEAS-2B cells were indirectly stained with anti-human IL-1R8 primary antibody or corresponding isotypic IgG control antibody (Santa Cruz Biotechnology Inc., Santa Cruz, CA, USA) followed by PE-labeled secondary antibody staining and analyzed by flow cytometry.

Supplementary Figure S2

Figure S2A. MTT assay on BEAS-2B cells.

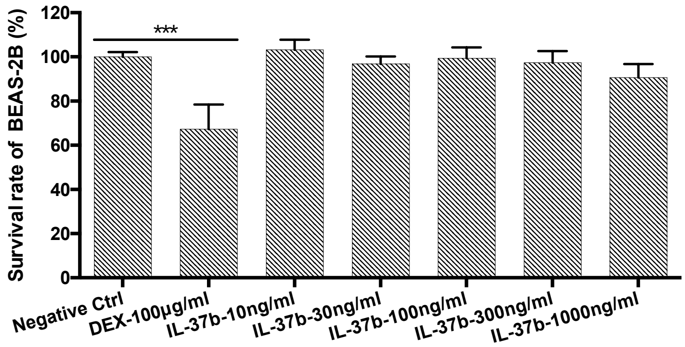

Figure S2B. 7-AAD viability staining assay on eosinophils.

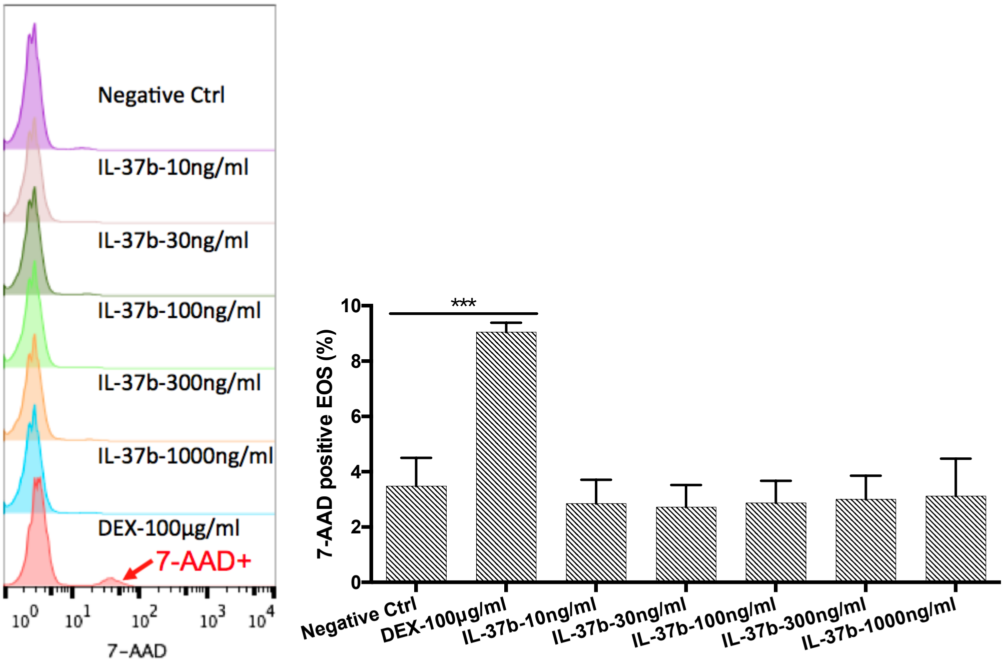

Supplementary Figure S2. The effect of IL-37b on cell proliferation and viability of BEAS-2B cells and eosinophils.

BEAS-2B cells and eosinophils were cultured separately in 96-well plate and treated with increasing concentration of IL-37b (10 – 1000 ng/ml) or DEX (100 μg/ml) for 20 h. After IL-37b treatment, (A) BEAS-2B cell proliferation was measured by MTT colorimetric assay. (B) Eosinophils were harvested for 7-AAD staining and cell viability was analyzed by flow cytometer. DEX, dexamethasone; MTT, methyl thiazolyl tetrazolium; 7-AAD, 7-amino-actinomycin D.

Supplementary Figure S3

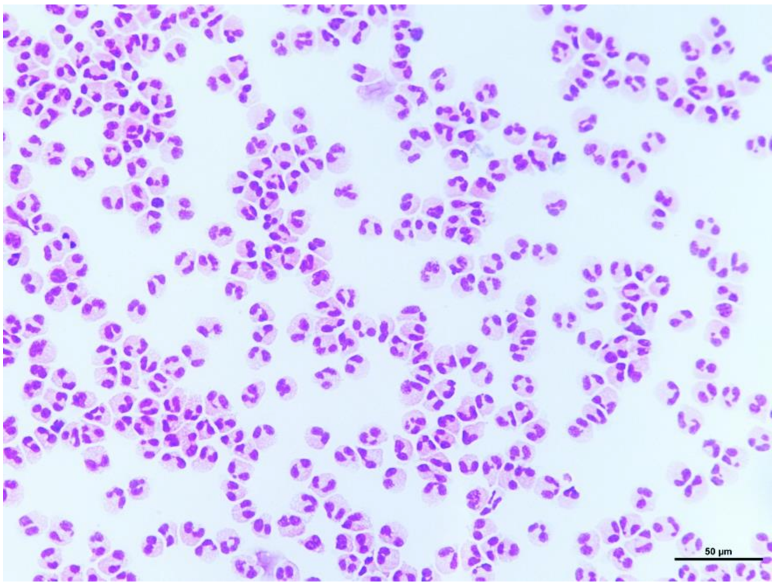

Supplementary Figure S3. Hemacolor rapid blood smear staining of co-cultured eosinophils.

The suspended co-cultured eosinophils were separated from adherent BEAS-2B cells. The purity of obtained eosinophils was checked by hemacolor rapid blood smear stain. Eosinophils were characterized by brick-red granules and bi-lobed nucleus in cytoplasma. The purity of eosinophils was more than 99% and no BEAS-2B cells were observed under the microscopic examination.

Supplementary Figure S4

Figure S4A. Timeline protocol of OVA-induced allergic asthma and IL-37b administration.

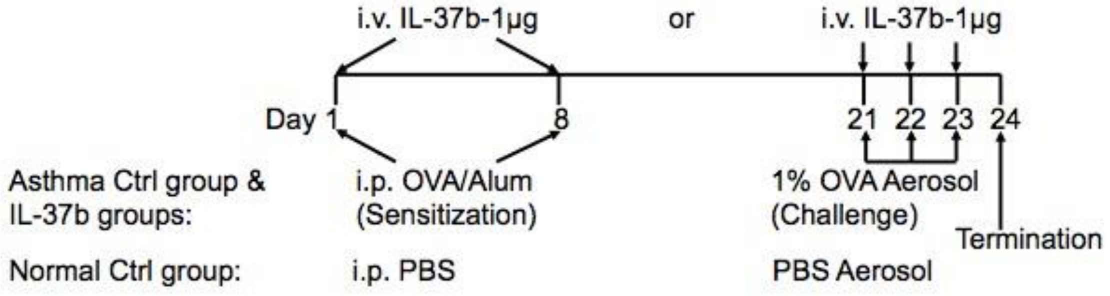

Figure S4B. The concentration of OVA-specific IgE in plasma.

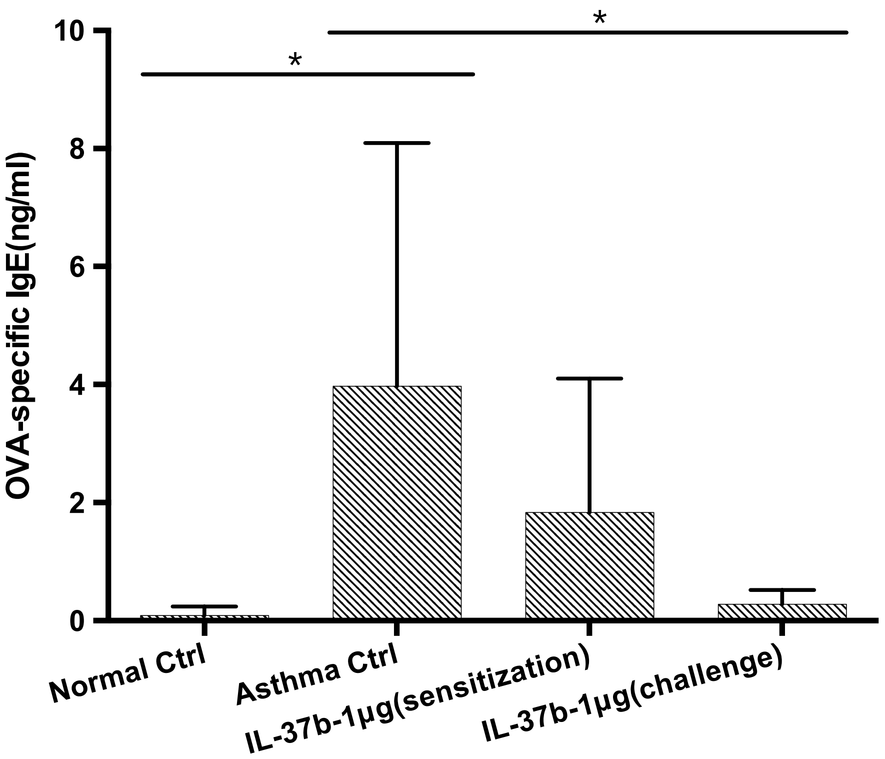

Figure S4C. Cytokines and chemokines expression in plasma.

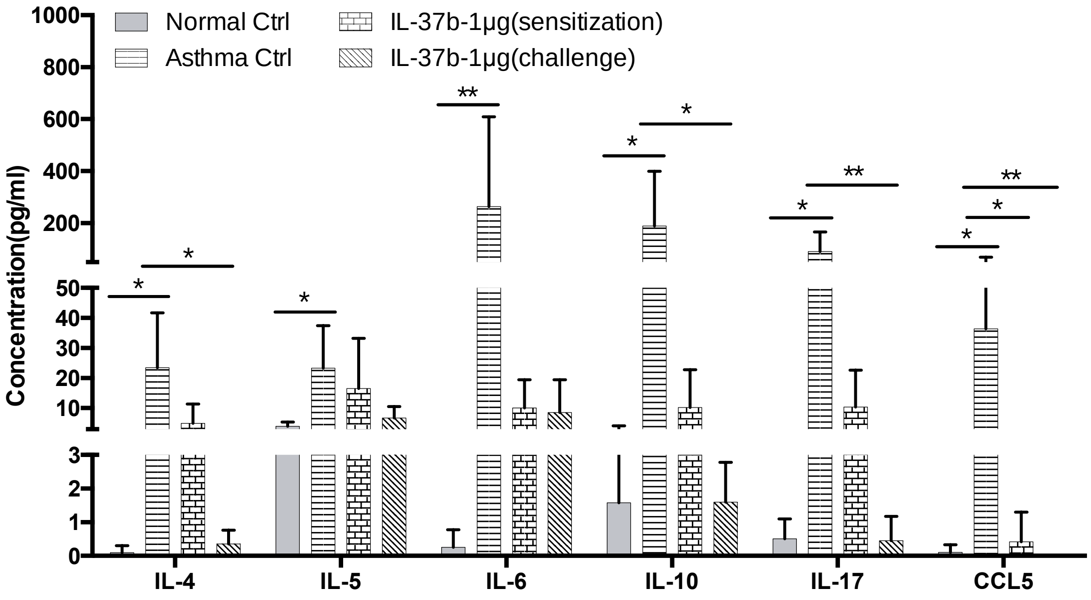

Figure S4D. Cytokines expression in BALF.

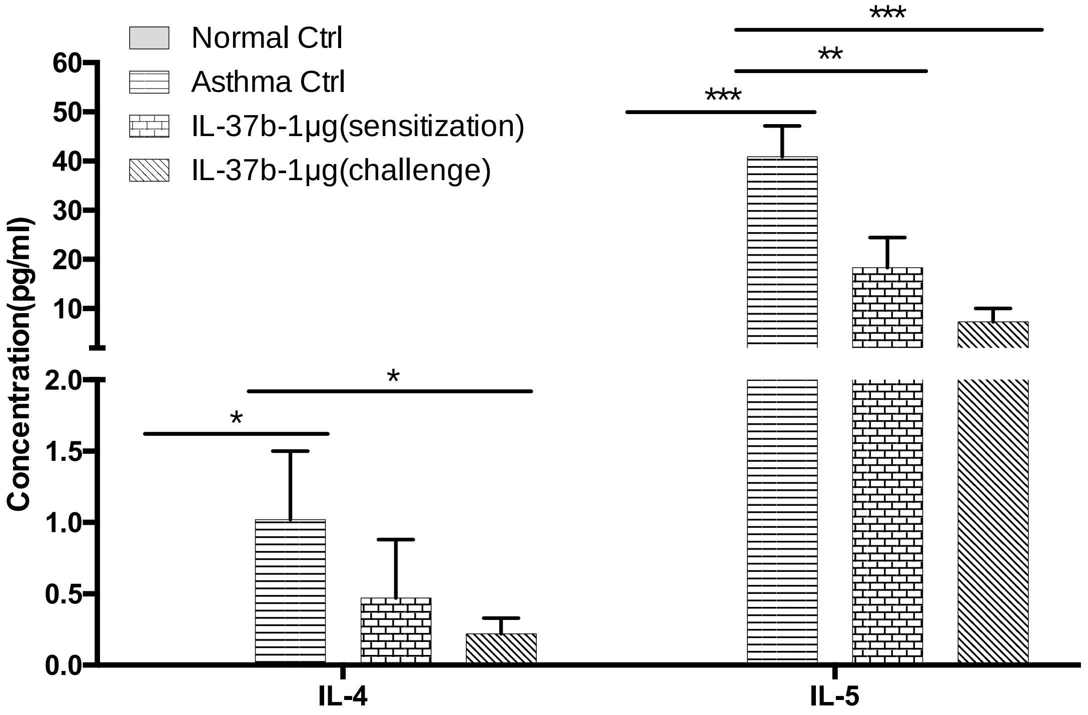

Figure S4E. Cytokines and chemokines expression in lung homogenate.

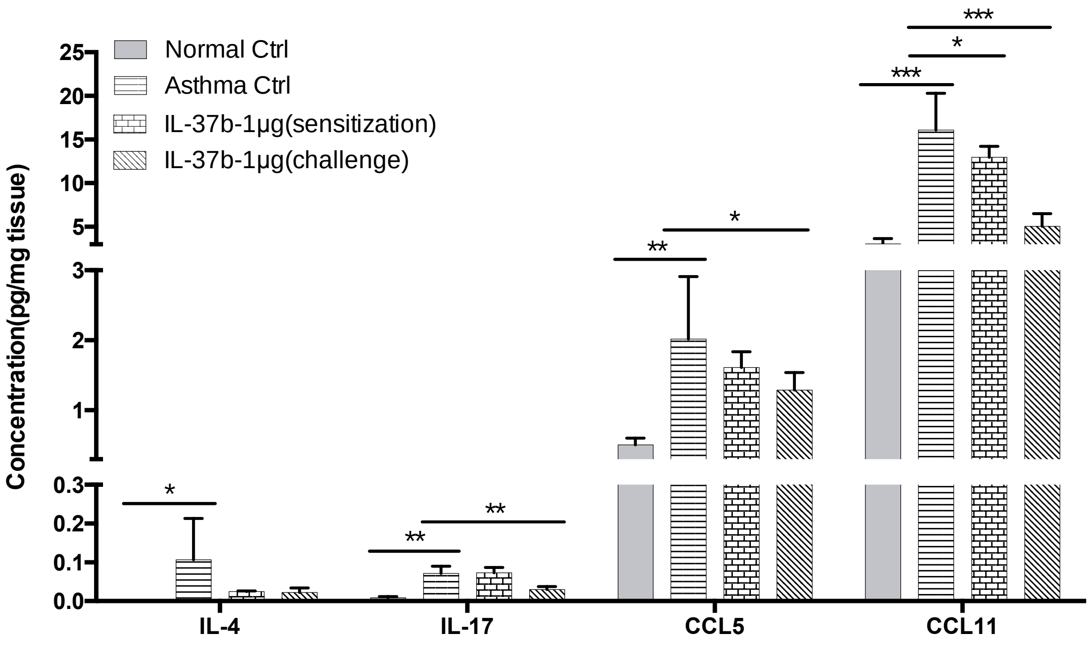

Figure S4F. The total cell number and differential cell counts in BALF.

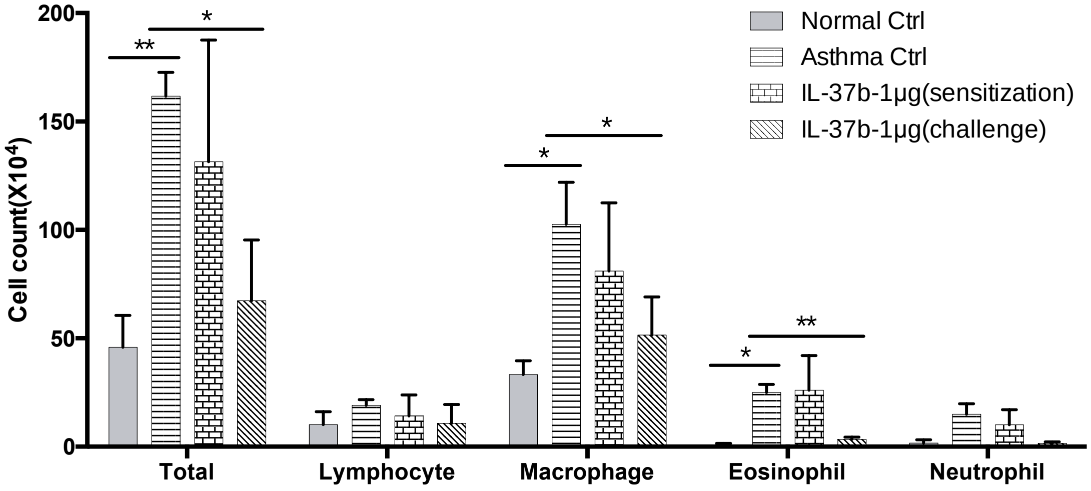

Figure S4G. Number of Eosinophils in BALF.

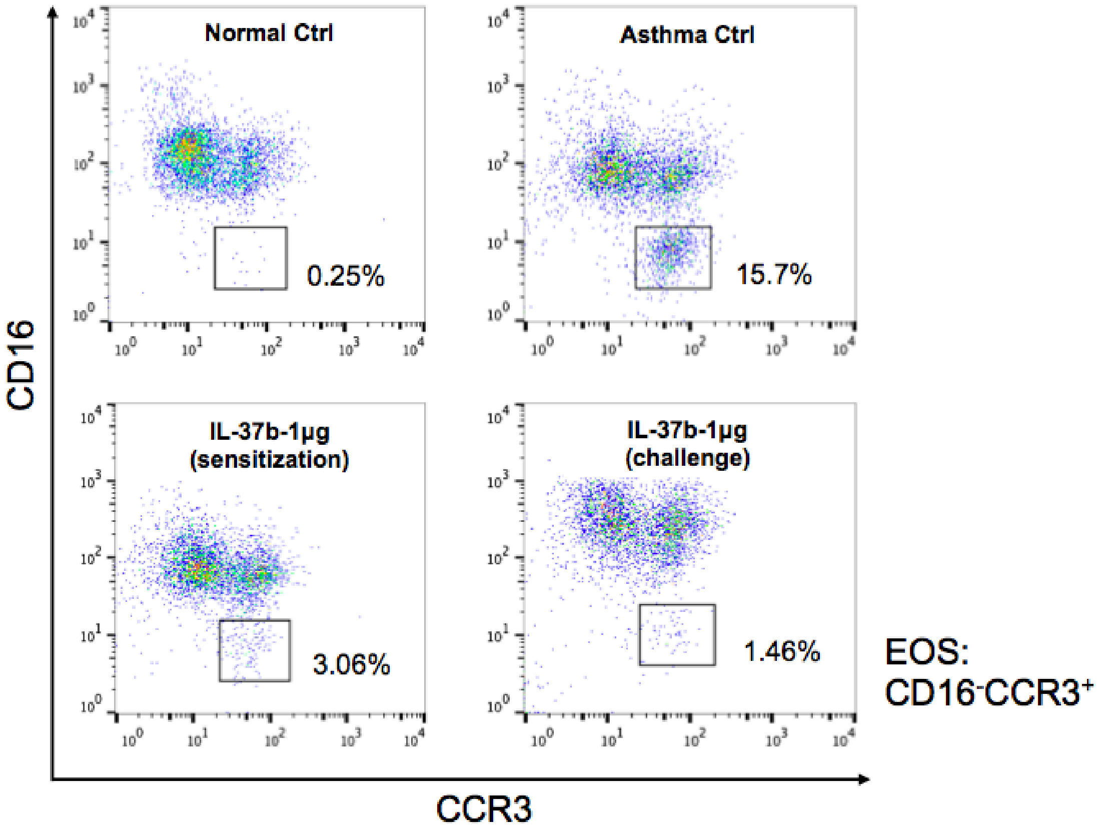

Figure S4H. The percentages of Treg cells in spleen and lung tissue.

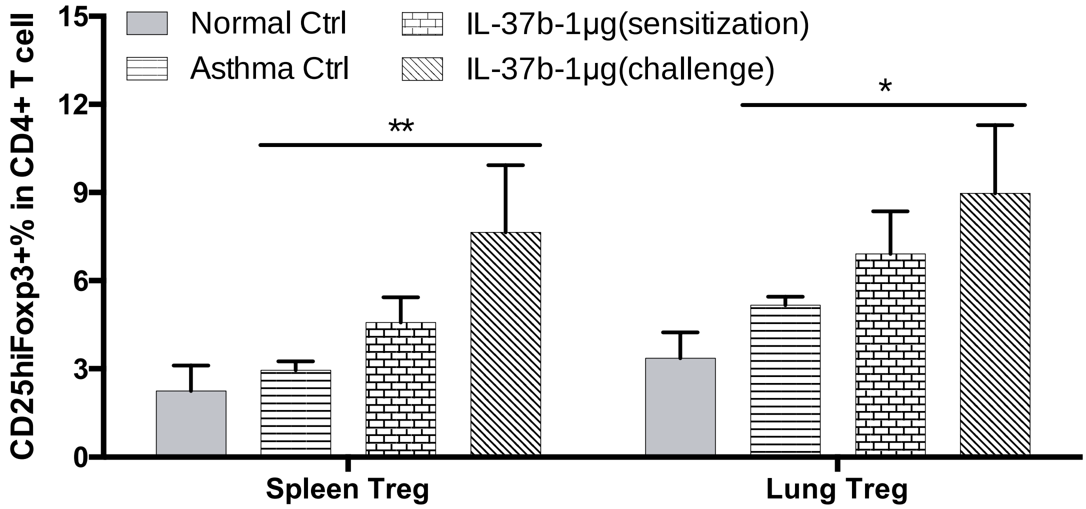

Figure S4I. Histological examination of lung section

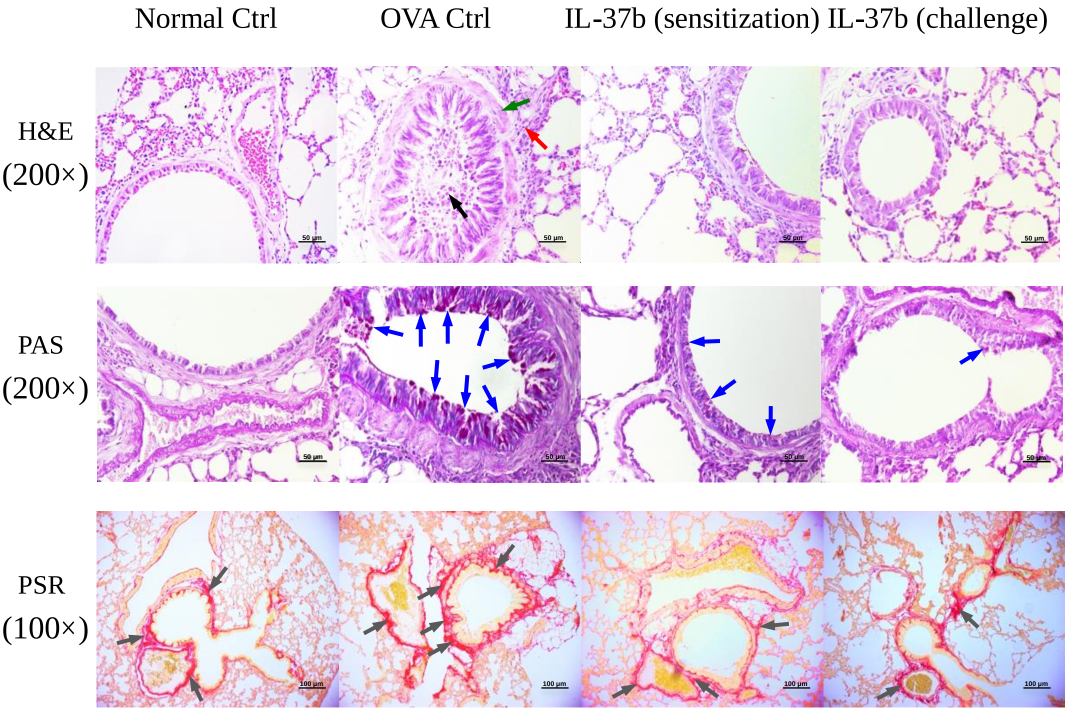

Supplementary Figure S4. *In vivo* regulatory activities of IL-37b on OVA-induced allergic airway inflammation.

BALB/c mice were sensitized with 20 μg OVA adsorbed to 1 mg Imject Alum (Thermo Fisher Scientific Inc., Rockford, IL, USA) or PBS by i.p. injection on days 1 and 8, with subsequent twice intranasal challenge with 1 % OVA or PBS aerosol on days 21, 22 and 23. Recombinant human IL-37b (1 μg) was i.v. injected before the sensitization or challenge. Mice were terminated 24 hours after the third challenge. (A) Timeline protocol of OVA-induced allergic asthma and IL-37b administration. (B) The concentration of OVA-specific IgE in plasma was determined. The protein concentrations of (C) IL-4, IL-5, IL-6, IL-10, IL-17, CCL5 in plasma (n = 6 per group), (D) IL-4, IL-5 in BALF (n = 3 per group) and (E) IL-4, IL-17, CCL5, CCL11 in lung homogenate (n = 3 per group) were also measured, $^{*}P < 0.05$, $^{**}P < 0.01$, $^{***}P < 0.001$ when compared between denoted groups. (F) The total cell number and differential cell counts in BALF were determined using Shandon Kwik-Diff Stains. (G) Eosinophils in BALF were identified as $CD16^{-}CCR3^{+}$ and (H) the percentages of regulatory T cells in spleen and lung tissue were identified as $CD25^{+}Foxp3^{+}$ in $CD4^{+}$ T cell using flow cytometry. (I) Representative staining with H&E, PAS (200× magnification) and Picro-sirius red/PSR (100× magnification) of lung sections are shown. The black, red and green arrows indicate increased mucus, inflammatory cell

infiltration and thickened airway wall, respectively. Goblet cells in lung tissue were indicated by blue arrow. Collagen deposition in lung tissue were stained by PSR staining and indicated by gray arrow. Bar charts were presented with mean ± SD. $^{*}P < 0.05$, $^{**}P < 0.01$, $^{***}P < 0.001$ when compared between denoted groups. OVA, ovalbumin; BALF, bronchoalveolar lavage fluid; Treg cells, regulatory T cells; H&E, hematoxylin and eosin; PAS, periodic acid-schiff; PSR, Picro-sirius red.

Supplementary Figure S5

Figure S5A. Plasma concentrations of HDM specific IgE and IgG1

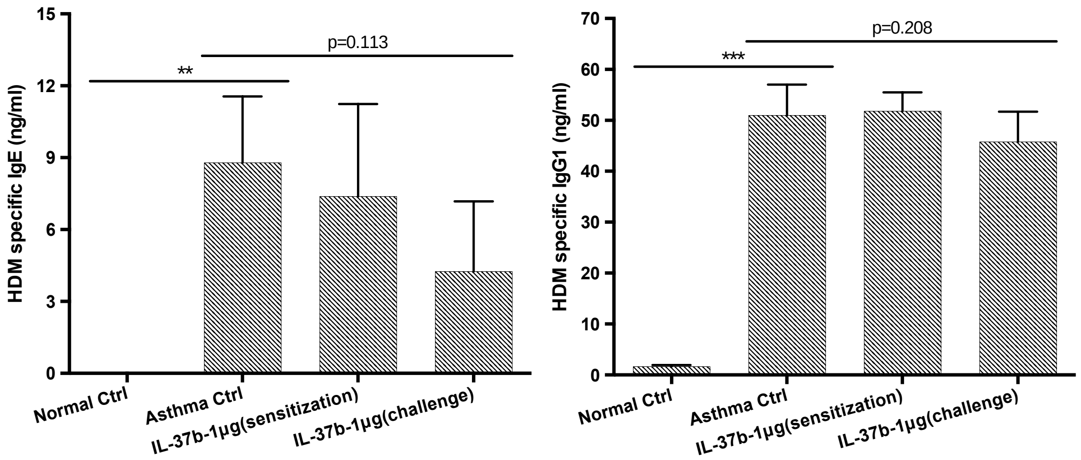

Figure S5B. Plasma concentrations of cytokines and chemokines

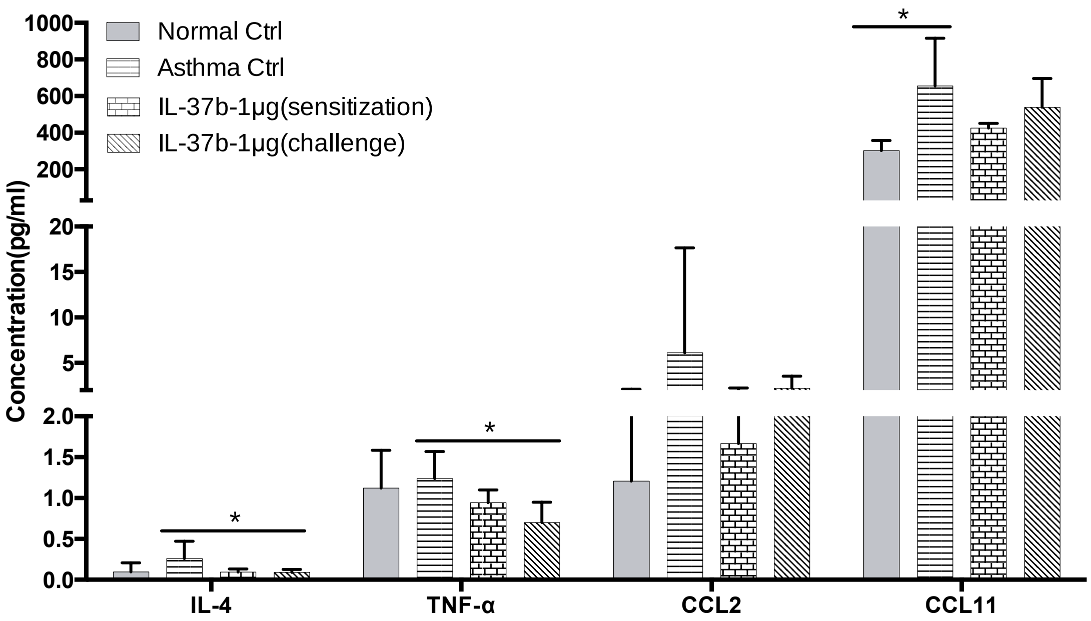

Figure S5C. BALF concentrations of cytokines

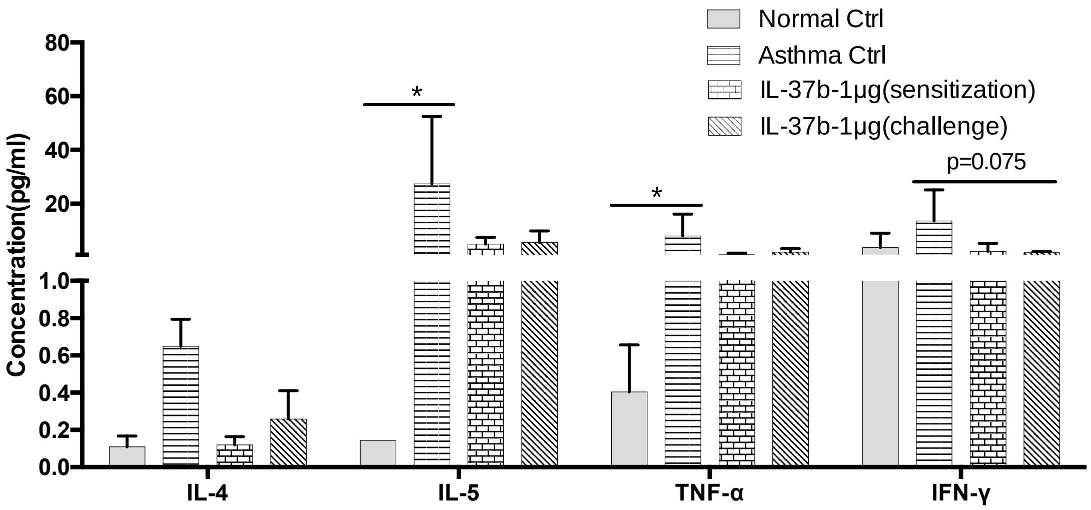

Figure S5D. Cytokines and chemokines expression in lung homogenate

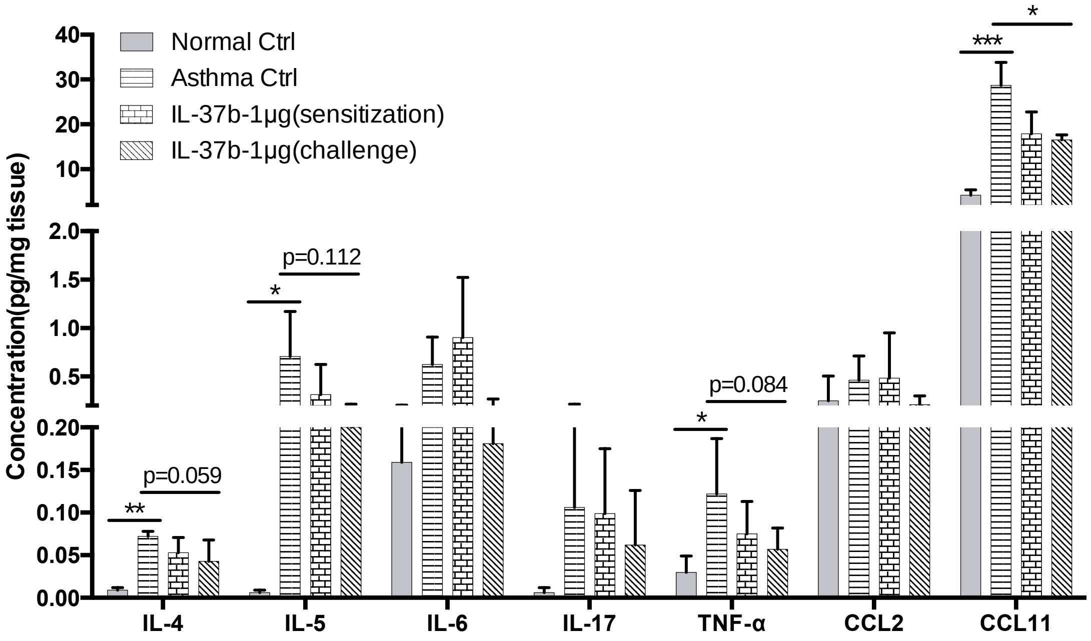

Figure S5E. Number of inflammatory cells in BALF

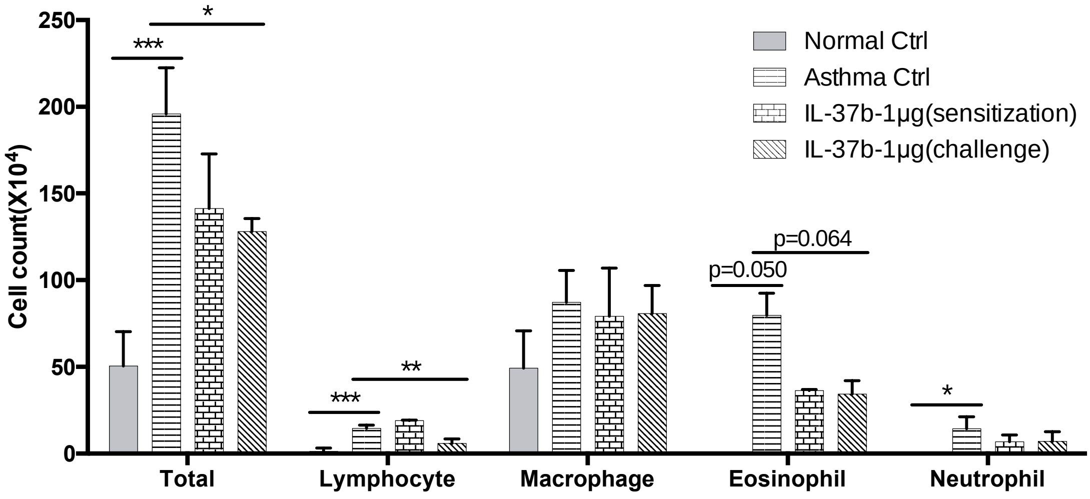

Figure S5F. Percentages of regulatory T cells in splenic tissue and lung tissue

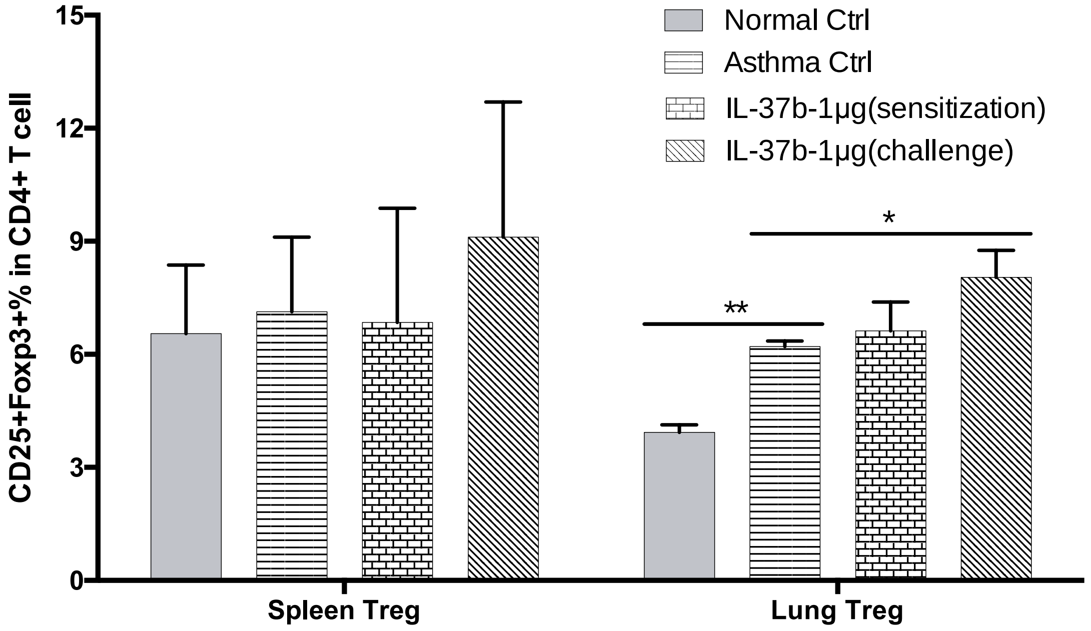

Figure S5G. Histological examination of lung section

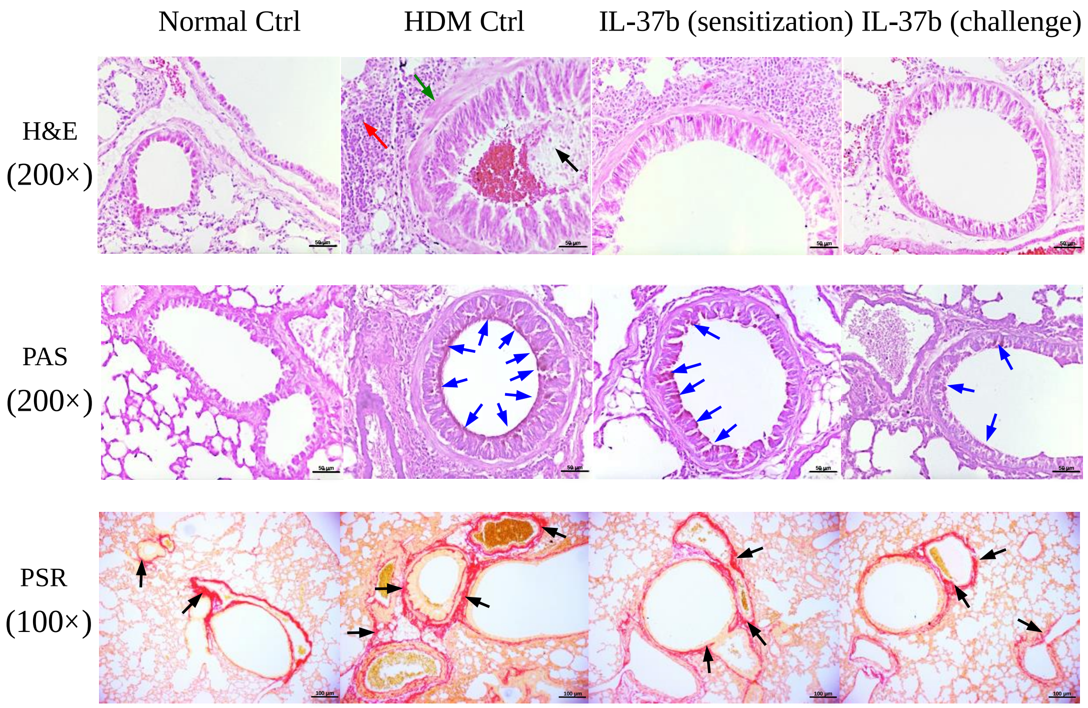

Supplementary Figure S5. The protective effect of IL-37b on HDM-induced asthmatic mice model.

BALB/c mice were sensitized with 1 μg HDM extract or PBS by intratracheal (i.t.) instillation on day 1, with daily challenge with 10 μg HDM or PBS by i.t. instillation on day 8 to day 12. Recombinant human IL-37b (1 μg) was i.v. injected just before the sensitization or challenge. Mice were terminated on day 17. (A) The concentration of HDM-specific IgE and IgG1 in plasma was determined. The protein concentrations of (B) IL-4, TNF-α, CCL2, CCL11 in plasma (n = 6 per group), (C) IL-4, IL-5, TNF-α, IFN-γ in BALF (n = 3 per group) and (D) IL-4, IL-5, IL-6, IL-17, TNF-α, CCL2, CCL11 in lung homogenate (n = 3 per group) were also measured. (E) The total cell number and differential cell counts in BALF were determined using Shandon Kwik-Diff Stains. (F) The percentages of regulatory T cells in spleen and lung tissue were identified as CD25^{+}Foxp3^{+} in CD4^{+} T cell using flow cytometry. (G) Representative staining with H&E, PAS (200× magnification) and PSR (100× magnification) of lung sections are shown. The black, red and green arrows indicate increased mucus, inflammatory cell infiltration and thickened airway wall, respectively. Goblet cells in lung tissue were indicated by blue arrow. Collagen deposition in lung tissue were stained by PSR staining and indicated by gray arrow. Bar charts were presented with mean ± SD. $^{*}P < 0.05$, $^{**}P < 0.01$, $^{***}P < 0.001$ when compared between denoted groups. HDM, house dust mite; BALF, bronchoalveolar

lavage fluid; Treg cells, regulatory T cells; H&E, hematoxylin and eosin; PAS, periodic acid-schiff; PSR, Picro-sirius red.

Supplementary Table S1. Primer sequences used in real-time qPCR

Genes		Primer Sequences (5'-3')
Human-*TNFα*	Forward	CCCAGGGACCTCTCTCTAATC
	Reverse	ATGGCTACAGGCTTGTCACT
Human-*IL-1β*	Forward	TGGCAATGAGGATGACTTGTTC
	Reverse	AAGGGAAAGAAGGTGCTCAGG
Human-*IL-6*	Forward	CCAGCTATGAACTCCTTCTC
	Reverse	GCTTGTTCCTCACATCTCTC
Human-*CCL2*	Forward	AAGATCTCAGTGCAGAGGCTCG
	Reverse	TTGCTTGTCCAGGTGGTCCAT
Human-*CXCL8*	Forward	ACTGAGAGTGATTGAGAGTGGAC
	Reverse	ACAACCCTCTGCACCCAGTT
Human-*BOLA2B*	Forward	CTTCAGAGACACAGGCTGGT
	Reverse	TGCAGATCCCAGTCCCTCAT
Human-*CAMP*	Forward	GGATGCTAACCTCTACCGCC
	Reverse	GGCACACTGTCTCCTTCACT
Human-*DPM3*	Forward	GGAGCTGCCCTTGTCCTG
	Reverse	GATAGCCCACAGTGCCCAG
Human-*ELOB*	Forward	AGACCACCATCTTCACGGAC
	Reverse	GTCATCCTTGTACAGCCGCT
Human-*C4ORF48*	Forward	GGACTAGAGGCTCGCTGGG
	Reverse	CGCCAGACTCAGCAGCAG
Human-*S100A9*	Forward	GCTGGTGCGAAAAGATCTGC
	Reverse	TGAACTCCTCGAAGCTCAGC
Human-*TFF3*	Forward	TTGGTGTTTCAAGCCCCTG
	Reverse	CGGGAGCAAAGGGACAGAAA
Human-*NPIPB15*	Forward	TGGGTTTCTGAGACGTGACA
	Reverse	AGCCCTTTCTGTCTACTGCG
Human-*PYCARD*	Forward	GAGAACCTGACCGCCGAG
	Reverse	CGTAGGTCTCCAGGTAGAAGC
Human-*GAPDH*	Forward	ATGGGGAAGGTGAAGGTCG
	Reverse	GGGGTCATTGATGGCAACAATA
